# Microarray Analysis of Novel Candidate Genes Responsible for Glucose-Stimulated Insulin Secretion in Mouse Pancreatic β Cell Line MIN6

**DOI:** 10.1371/journal.pone.0061211

**Published:** 2013-04-03

**Authors:** Eiji Yamato, Fumi Tashiro, Jun-ichi Miyazaki

**Affiliations:** Division of Stem Cell Regulation Research, Osaka University Graduate School of Medicine, Suita, Osaka, Japan; College of Tropical Agriculture and Human Resources, University of Hawaii, United States of America

## Abstract

Elucidating the regulation of glucose-stimulated insulin secretion (GSIS) in pancreatic islet β cells is important for understanding and treating diabetes. MIN6 cells, a transformed β-cell line derived from a mouse insulinoma, retain GSIS and are a popular *in vitro* model for insulin secretion. However, in long-term culture, MIN6 cells' GSIS capacity is lost. We previously isolated a subclone, MIN6 clone 4, from the parental MIN6 cells, that shows well-regulated insulin secretion in response to glucose, glybenclamide, and KCl, even after prolonged culture. To investigate the molecular mechanisms responsible for preserving GSIS in this subclone, we compared four groups of MIN6 cells: Pr-LP (parental MIN6, low passage number), Pr-HP (parental MIN6, high passage number), C4-LP (MIN6 clone 4, low passage number), and C4-HP (MIN6 clone 4, high passage number). Based on their capacity for GSIS, we designated the Pr-LP, C4-LP, and C4-HP cells as “responder cells.” In a DNA microarray analysis, we identified a group of genes with high expression in responder cells (“responder genes”), but extremely low expression in the Pr-HP cells. Another group of genes (“non-responder genes”) was expressed at high levels in the Pr-HP cells, but at extremely low levels in the responder cells. Some of the responder genes were involved in secretory machinery or glucose metabolism, including *Chrebp*, *Scgn*, and *Syt7*. Among the non-responder genes were *Car2*, *Maf*, and *Gcg*, which are not normally expressed in islet β cells. Interestingly, we found a disproportionate number of known imprinted genes among the responder genes. Our findings suggest that the global expression profiling of GSIS-competent and GSIS-incompetent MIN6 cells will help delineate the gene regulatory networks for insulin secretion.

## Introduction

MIN6 cells, which were isolated from an insulinoma of a transgenic mouse expressing the SV40 T antigen in pancreatic islet β cells, retain some features of differentiated pancreatic β cells [1]. However, long-term culture of MIN6 cells results in the loss of their insulin secretory capacity in response to glucose [2]–[4]. Several reports have described the isolation of glucose-responsive and -unresponsive MIN6 cell subclones, and these subclones have been used to identify genes associated with glucose-stimulated insulin secretion (GSIS) [5], [6]. However, these reports did not rule out the possibility that this differential gene expression was attributable simply to clonal variation. In addition, further analyses of the phenotypic stability of the glucose-responsive MIN6 subclones during long-term culture have not been performed.

Gene expression profile analysis has been performed to compare MIN6 cells after short-term and long-term culture [4]. In addition, comparative proteomic analyses of the cell lysates [7] and the conditioned media [8] were used to investigate the relative changes in the proteome that accompany the loss of GSIS over time in culture. Recently, impairment of glucose and lipid oxidation was suggested to be involved in the loss of GSIS in high passage MIN6 cells [9]. However, these studies did not verify that the differentially expressed genes and proteins were directly involved in the mechanisms for GSIS.

In the present study, we used a MIN6 subclone, designated MIN6 clone 4, which retains GSIS even after long-term culture. To identify the genes involved in the maintenance of GSIS capacity, we compared the gene expression profiles among parental MIN6 cells after short-term culture (Pr-LP) and long-term culture (Pr-HP) and of MIN6 clone 4 cells after short-term culture (C4-LP) and long-term culture (C4-HP). The results revealed one group of genes whose expression was high in well-regulated (i.e., low passage number) parental MIN6 cells and MIN6 clone 4 cells, but extremely low in the dysregulated (high passage number) parental MIN6 cells. Another group of genes was expressed at extremely low levels in the well-regulated MIN6 cells, but at high levels in the dysregulated ones. We discuss the roles of these differentially expressed genes in insulin secretion.

## Materials and Methods

### Culture of MIN6 parental cells and subclone

MIN6 cells, which we have previously established [1], were maintained in Dulbecco's modified Eagle's medium containing 25 mM glucose, 13% heat-inactivated fetal bovine serum, 0.1 mM 2-mercaptoethanol, 100 units/ml penicillin, and 0.05 mg/ml streptomycin in humidified 5% CO_2_ at 37°C [1]. MIN6 clone 4 cells are a subclone isolated from low-passage-number parental MIN6 cells by the limiting dilution method. This subclone was maintained in the same culture conditions as the parental cells, and retained good GSIS even after 6 months of continuous culture. For the low-passage-number parental MIN6 cells (Pr-LP), we used cells passaged 17–20 times; for the high-passage-number MIN6 cells (Pr-HP), we used cells passaged 35–40 times. Seventeen to 20 passages were also used for the low-passage-number MIN6 clone 4 cells (C4-LP), and the high-passage ones (C4-HP) were used after 40 to 50 passages. For all of the following methods sections, the parental MIN6 cells and their subclone were treated identically.

### Measurement of insulin secretion and insulin content

MIN6 cells were cultured in 24-well plates for 4 days. Prior to the insulin secretion assay, the cells were starved in Krebs Ringer solution containing 0.1% bovine serum albumin (BSA) with 3 mM glucose for 30 min, and the wells were washed twice with the same buffer. The cells were then incubated in Krebs Ringer Solution with 3, 8, 15, or 25 mM glucose, 100 µM glybenclamide+3 mM glucose, or 30 mM KCl+3 mM glucose for 1 hr.

Insulin secreted into the medium and contained in the MIN6 cells was measured. For the secreted insulin, medium was collected and the insulin measured using an ELISA kit (Cat.#10-1250-01; Mercodia, Uppsala, Sweden). To normalize the amount of secreted insulin to the protein content of each well, the cells in each well were lysed with RIPA buffer, and the protein concentration of the cell lysates was measured by the Bradford method (Cat.#500-0006; Bio-Rad, Hercules, CA). To measure the insulin content of MIN6 cells, we lysed cells in a different set of wells with acid-ethanol, and centrifuged the cell extracts. The amount of insulin in the supernatants was assayed using the ELISA kit. Statistical analysis was performed by Student′s *t*-test.

### DNA microarray analyses

Total RNA was extracted from MIN6 cells using Trizol reagent (Invitrogen, Carlsbad, CA) and subjected to double-strand cDNA synthesis using the Superscript Choice system (Cat.#18090-019; Invitrogen) and the T7-(dT)_24_ reverse transcription primer (Cat.#72-1591-01; Amersham Biosciences, Piscataway, NJ). Synthesis of biotin-labeled cRNA was carried out by *in-vitro* transcription using the Bio Array RNA Transcript Labeling Kit (Cat.#900182; Affymetrix, Santa Clara, CA) according to the manufacturer's instructions. The biotin-labeled cRNA was purified using RNeasy spin columns (Cat.#74106; Qiagen GmbH, Hilden, Germany) and fragmented in a reaction mixture. The biotin-labeled and fragmented cRNA was hybridized to the murine genome U74 version 2 GeneChip array (Affymetrix), incubated, and washed according to the manufacturer's instructions. The GeneChip arrays were then scanned with a Gene Array Scanner (Hewlett-Packard, Santa Clara, CA) and analyzed by GeneChip 5.1 software (Affymetrix). The microarray dataset has been deposited in NCBI's Gene Expression Omnibus and is accessible through GEO Series accession number GSE43774.

### Quantitative RT-PCR

Total RNA was extracted from MIN6 cells by the acid guanidinium-phenol-chloroform (AGPC) method and subjected to cDNA synthesis using ReverTra Ace α (Cat.#FSK-101; Toyobo, Tokyo, Japan). Quantitative RT-PCR analysis was carried out using SYBR Premix Ex Taq (Cat.#RR041A; Takara, Otsu, Japan). The reaction was performed with 1 µl cDNA per 25 µl reaction in a 7300 Real-Time PCR System (Applied Biosystems, Foster City, CA) under the following thermal cycling conditions: 95°C for 10 sec followed by 40 cycles at 95°C for 5 sec and 60°C for 31 sec. The relative expression levels of the target genes were normalized to that of *Rpl32*. Statistical analysis was performed by Student′s *t*-test. Primer sequences are listed in **Table S1**.

### Immunocytochemistry and FACS (fluorescence-activated cell sorting) analysis

For immunocytochemistry, the MIN6 cells were washed with PBS and fixed in 4% paraformaldehyde for 10 min, rinsed with PBS, incubated for 5 min in PBS with 1% Triton X-100, washed again with PBS, and incubated in blocking reagent (Blocking One, Cat.#03953-95; Nacalai Tesque, Kyoto, Japan). The samples were incubated with rat anti-CD24 antibody (Cat.#14-0241-81; eBioscience, San Diego, CA) for 1 hr at room temperature, washed with PBS, and then incubated with Alexa Fluor 488-conjugated anti-rat IgG (Cat.#A-11006; Molecular Probes, Eugene, OR) for 1 hr at room temperature. For FACS analysis, the cells were washed twice with PBS and then suspended in SM buffer (HEPES-buffered saline with 0.1% sodium azide and 1% BSA). CD24 staining was performed at 4°C for 30 min with rat anti-CD24 antibody, followed by incubation with Alexa Fluor 488-conjugated anti-rat IgG. FACS analyses were performed with a FACScan (Becton Dickinson, Franklin Lakes, NJ) using the CellQuest (Becton Dickinson) evaluation program.

### *In situ* hybridization

Part of the mouse *Hepacam2* cDNA (NM_178899; sequence position 58–699) was subcloned into the pGEMT-Easy vector (Cat.#A1360; Promega, Madison, WI) and used to generate sense and antisense RNA probes. Digoxigenin (DIG)-labeled RNA probes were prepared with the DIG RNA Labeling Mix (Cat.#1277073; Roche, Mannheim, Germany) according to the manufacturer's instructions. *In situ* hybridization was performed according to the protocol of Genostaff (Tokyo, Japan). In brief, a pancreas of a C57BL/6J mouse was dissected out after perfusion and fixation with Tissue Fixative (Genostaff, Tokyo, Japan), embedded in paraffin, and sectioned at 6 µm. The tissue sections were de-waxed and fixed with 4% paraformaldehyde in PBS. The sections were then hybridized with sense and antisense *Hepacam2* probes. After treatment with blocking reagent in TTBS (10 mM Tris-HCl, pH 7.6, 137 mM NaCl, and 0.1% Tween 20) for 30 min, the sections were incubated with anti-DIG alkaline phosphatase (AP) conjugate (Cat.#1093274; Roche) diluted 1∶1000 with TTBS for 2 hr at room temperature, washed twice with TTBS, and then incubated in 100 mM Tris-HCl, pH 9.5, 100 mM NaCl, 50 mM MgCl_2_, and 0.1% Tween 20 for 30 min. Coloring reactions were performed with NBT/BCIP solution (Cat.#B6404; Sigma, St. Louis, MO) overnight, and the sections were then washed with PBS and counterstained with Kernechtrot stain solution (Cat.#40872; Mutoh Chemical Co., Tokyo, Japan).

### Bisulfite sequencing

Bisulfite treatment of the genomic DNA isolated from Pr-LP, Pr-HP, C4-LP, and C4-HP MIN6 cells was performed using the EpiTect Bisulfite Kit (Cat.#59104; Qiagen) according to the manufacturer's instructions. The CpG islands in the first intron of the *Plagl1* gene (33 CpGs) and those in the DMR region of the *Dlk1* gene (24 CpGs) were chosen for analysis. The primers for the *Plagl1* gene were: forward, 5′-GGGTAGGTAAGTAGTGATAA-3′; reverse, 5′-CCTAAAACACCAAAATAACA-3′ [10]. The primers for the *Dlk1* gene were: forward, 5′-GATTAGTGATTTATAATTTGTGTTTTGGTT-3′; reverse, 5′-AAACTCACCTAAATATACTAAAAACAAATA-3′ [11]. The PCR products were cloned into pBluescript and sequenced.

### Statistical analysis

Results are presented as the mean ± SD. Statistical analyses were carried out by Student's *t*-test except for that of the distribution of the percentages of methylated CpGs in the *Plagl1* locus, which was carried out by the F-test. A value of *P*<0.05 was considered statistically significant.

## Results

### Insulin secretion and insulin content

**Figure 1:**
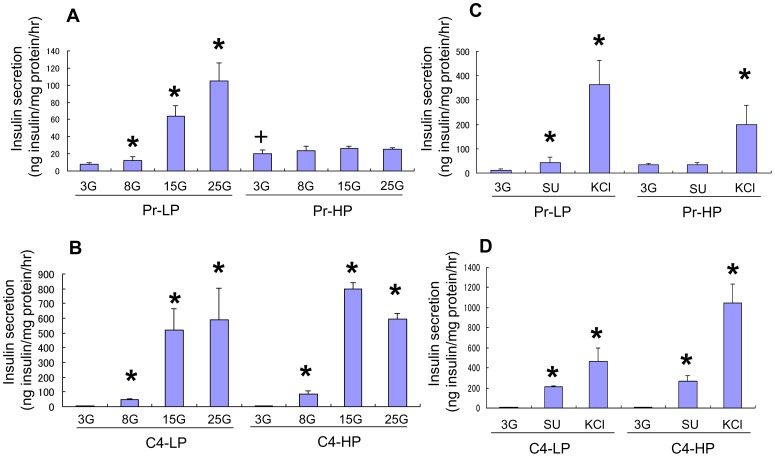
Insulin secretion from MIN6 cells. Insulin secretion from Pr-LP, Pr-HP, C4-LP, and C4-HP MIN6 cells stimulated with 3 mM (3G), 8 mM (8G), 15 mM (15G), or 25 mM (25G) glucose (A, B), 100 nM glybenclamide (SU), or 30 mM KCl (C, D). Pr-HP cells showed higher basal insulin secretion at 3 mM glucose compared with Pr-LP cells, but their insulin secretion did not increase further at higher glucose concentrations or with the addition of glybenclamide or KCl, whereas both C4-LP and C4-HP MIN6 cells showed a better insulin secretory response to glucose and glybenclamide than Pr-LP cells. Values are means ± SD and n = 5–6. *P<0.05 v.s. insulin secretion at 3 mM glucose.+*P*<0.05 v.s. insulin secretion of Pr-LP, C4-LP, and C4-HP cells at 3 mM glucose by Student's *t*-test.

**Figure 2:**
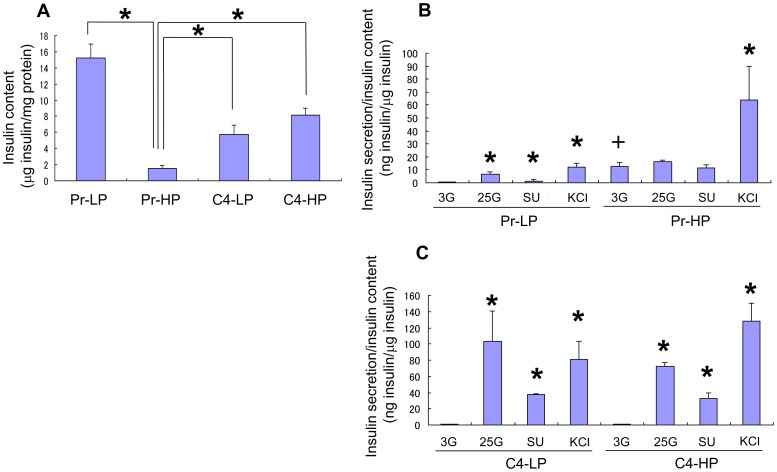
Insulin content and secretion of MIN6 cells. Insulin content of Pr-LP, Pr-HP, C4-LP, and C4-HP MIN6 cells (A). The insulin content of Pr-HP cells was lower than that of Pr-LP, C4-LP, or C4-HP cells. Values are means ± SD and n = 5–6. *P<0.05. Insulin secretion/insulin content from Pr-LP, Pr-HP, C4-LP, and C4-HP MIN6 cells stimulated with 3 mM (3G), 25 mM (25G) glucose, 100 nM glybenclamide (SU), or 30 mM KCl (B, C). Values are means ± SD and n = 5–6. *P<0.05 v.s. insulin secretion at 3 mM glucose.+*P*<0.05 v.s. insulin secretion of Pr-LP, C4-LP, and C4-HP cells at 3 mM glucose by Student's *t*-test.

Insulin secretory capacity was compared by static incubation among parental MIN6 cells at 17–20 passages (Pr-LP) and 35–40 passages (Pr-HP) and MIN6 clone 4 cells at 17–20 passages (C4-LP) and 40–50 passages (C4-HP) (**Figure 1** **and** **2**). Pr-HP cells showed a higher basal insulin secretion at 3 mM glucose than did the other groups, but the insulin secretion did not increase with higher glucose concentrations or the addition of glybenclamide (SU) or KCl, in contrast with the other three groups (**Figure 1** **,** **2B, and 2C**). In addition, the insulin content of the Pr-HP cells was much lower than that of the Pr-LP, C4-LP, or C4-HP cells (**Figure 2A**). Therefore, we designated the C4-LP, C4-HP, and Pr-LP cells as “responder” cells and the Pr-HP cells as “non-responder” cells.

Furthermore, both C4-LP and C4-HP cells showed a better insulin secretory response to glucose and glybenclamide than did the Pr-LP cells (**Figure 1**), suggesting that the MIN6 clone 4 cells maintained stable regulatory mechanisms for insulin secretion. Interestingly, the insulin secretion induced with KCl was even higher in the C4-HP cells than in the C4-LP cells (**Figure 1D** **and** **2C**).

### DNA Microarray analysis

To identify genes involved in the regulation of the insulin secretory pathway, a comparative DNA microarray analysis was performed, using the Pr-LP, Pr-HP, C4-LP, and C4-HP cells. The results revealed one group of genes, which we call “responder genes,” that were highly expressed in the responder cells (Pr-LP, C4-LP, and C4-HP), but only weakly in the non-responder cells (Pr-HP). A different group of genes (“non-responder genes”) was highly expressed in the Pr-HP cells, but only weakly in the Pr-LP, C4-LP, and C4-HP cells. To compare the responder and non-responder results, we compared the mean value of the gene expression levels in the Pr-LP, C4-LP, and C4-HP cells with the expression level in the Pr-HP cells. Genes that showed a more than 5-fold difference in expression level are listed in **Table 1** (40 responder genes) and **Table 2** (41 non-responder genes). An extended list, including all the genes that showed a more than 3-fold difference, is provided in **Table S2** (60 responder and 62 non-responder genes). Genes that were differentially expressed among Pr-LP, C4-LP, and C4-HP cells are also listed in **Table S3, S4, S5**.

**Table 1:** Genes preferentially expressed in responder MIN6 cells.

Gene name	Mean value for Pr-LP, C4-LP, and C4-HP*	Pr-HP*	Fold change
*Tmem59l*	3306.8	3.8	876.5
*Mlxipl*	750.0	<1.0	>750.0
*Scgn*	2686.6	10.3	261.6
*Tmed6*	107.7	<1.0	>107.7
*Plagl1*	4616.1	114.0	40.5
*Hepacam2*	2767.3	73.5	37.6
*Dlk1*	11245.0	323.7	34.7
*Rps6kb1*	119.0	5.4	22.2
*Syt7*	1327.9	65.9	20.1
*Slc29a4*	1531.1	78.9	19.4
*Cd24a*	4806.0	282.5	17.0
*Rpgr*	119.3	7.3	16.4
*Meg3*	2463.4	154.3	16.0
*Glul*	798.1	53.2	15.0
*1700019D03Rik*	210.7	15.2	13.9
*Gm2115*	607.8	44.8	13.6
*Nnat*	7113.3	637.7	11.2
*Ndn*	966.5	94.6	10.2
*Ly6e*	2512.2	250.9	10.0
*Gucy2c*	2118.9	217.6	9.7
*3830403N18Rik*	345.9	38.0	9.1
*Kctd12*	665.0	73.4	9.1
*Cd200*	508.3	61.7	8.2
*Th*	3705.1	477.8	7.8
*Amdhd2*	252.8	34.9	7.2
*Blnk*	2957.6	414.8	7.1
*Celsr2*	221.2	31.6	7.0
*Cplx2*	852.7	122.2	7.0
*Tmod2*	530.2	77.1	6.9
*Cdhr1*	1980.3	288.5	6.9
*Ppp1r3d*	495.8	73.8	6.7
*Pparg*	140.7	21.4	6.6
*Alcam*	278.1	42.5	6.6
*Unc80*	579.0	93.3	6.2
*Epb4.1l4b*	1667.5	270.6	6.2
*Akr1c14*	559.7	91.6	6.1
*A830039N20Rik*	285.8	48.3	5.9
*Mirg*	240.4	41.6	5.8
*Fgf12*	414.6	73.3	5.7
*Slc44a1*	218.2	39.1	5.6

* Raw values of expression intensities measured by Affymetrix arrays.

**Table 2:** Genes preferentially expressed in non-responder MIN6 cells.

Gene name	Pr-HP*	Mean value for Pr-LP, C4-LP, and C4-HP*	Fold change
*Cd68*	2507.1	79.8	31.4
*Car2*	2841.2	93.0	30.6
*Stmn2*	1994.6	65.5	30.5
*Maf*	536.5	22.0	24.4
*Il13ra1*	237.9	11.1	21.4
*Perp*	998.0	52.7	19.0
*Sh3bgrl2*	512.9	30.6	16.8
*Fgfr2*	1035.3	66.6	15.6
*Pqlc3*	2170.1	171.1	12.7
*Prr5l*	969.2	81.1	12.0
*Il4*	217.4	19.9	10.9
*Ptgs2*	195.8	18.2	10.7
*Scel*	316.3	30.5	10.4
*Lpl*	1474.6	160.6	9.2
*Rbfox1*	491.0	54.5	9.0
*Lcp2*	710.5	86.2	8.2
*Tbcel*	397.9	50.7	7.9
*Glyat*	204.5	26.9	7.6
*Tmcc3*	879.6	117.5	7.5
*Basp1*	153.9	21.5	7.2
*Pcdh7*	357.1	52.0	6.9
*Ppap2a*	1835.0	271.5	6.8
*Edil3*	1365.9	207.3	6.6
*S100a10*	4357.1	661.4	6.6
*Sox11*	1180.7	188.5	6.3
*Rpp25*	346.5	55.8	6.2
*Enpep*	757.0	123.7	6.1
*Bach2*	216.5	35.9	6.0
*1810011O10Rik*	721.4	120.3	6.0
*Gcg*	8993.3	1553.4	5.8
*Eif2ak4*	634.5	110.2	5.8
*Sash1*	566.6	99.7	5.7
*Hspbp1*	603.6	106.8	5.7
*Oat*	1753.3	327.6	5.4
*Ezr*	1299.8	243.9	5.3
*Gnai1*	1080.2	202.5	5.3
*Gnptab*	1234.2	231.4	5.3
*Fam132a*	984.7	185.9	5.3
*Zfp185*	669.7	126.7	5.3
*Ctsb*	199.4	39.5	5.1
*Hlf*	379.4	75.8	5.0

* Raw values of expression intensities measured by Affymetrix arrays.

**Figure 3:**
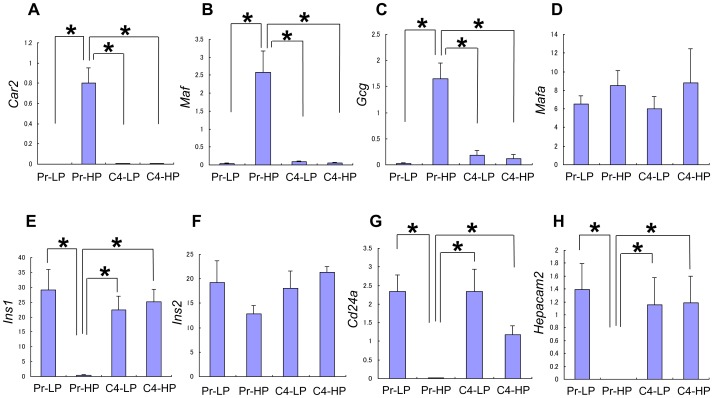
Quantitative RT-PCR analysis. Expression of the *Car2* (A), *Maf* (B), *Gcg* (C), *Mafa* (D), *Ins1* (E), *Ins2* (F), *Cd24a* (G), and *Hepacam2* (H) genes in Pr-LP, Pr-HP, C4-LP, and C4-HP MIN6 cells was examined by quantitative RT-PCR. *Car2*, *Maf*, and *Gcg* showed a non-responder gene expression pattern that was consistent with the DNA microarray analysis. *Ins2* and *Mafa* did not show significant differences in expression among the four groups of MIN6 cells, whereas *Ins1* was revealed to be a responder gene. *Cd24a* and *Hepacam2* showed a responder gene expression pattern that was consistent with the DNA microarray analysis. Values are means ± SD and n = 4–5. *P<0.05.

The expression pattern of some responder and non-responder genes was examined by quantitative RT-PCR (**Figure 3** **; Figure S1**). The four responder genes chosen, *Chrebp*, *Syt7*, *Cplx2*, and *Scgn* (**Table 1**), were highly expressed in the responder cells, but only weakly in Pr-HP cells (**Figure S1A-D**). These genes are known to be expressed in islet β cells and probably have some roles in the regulation of GSIS in β cells (see Discussion). The three non-responder genes chosen, *Car2*, *Maf*, and *Gcg* (**Table 2**), were highly expressed in Pr-HP cells, but only weakly in the responder cells (**Figure 3A-C**). These genes are known to be expressed in the pancreas, but not in islet β cells. Thus, the Pr-HP cells can express genes that are not normally expressed in islet β cells.

Genes encoding β-cell-related transcription factors are listed in **Table 3**. *Pdx1*, *Nkx2-2*, *Nkx6-2*, *Foxa2*, *Pax4*, and *Pax6* were not differentially expressed among the four groups of MIN6 cells, but *Neurod1* showed a 1.5-fold higher expression in the responder cells than in Pr-HP cells. Because the *Mafa* gene was not included in the U74 version 2 GeneChip array used in our analysis, we performed a quantitative RT-PCR analysis, which showed that *Mafa* gene expression did not differ significantly among the four MIN6 cell groups (**Figure 3D**).

**Table 3:** Expression levels of pancreas-related genes.

Gene Name	Pr-LP*	Pr-HP*	C4-LP*	C4-HP*	Mean**	Fold change***
*Pdx1*	701.4	519.5	591.7	570.0	621.0	0.84
*Nkx2-2*	2408.6	1395.5	1612.7	1501.1	1840.8	0.76
*Nkx6-2*	252.6	179.5	141.7	395.3	263.2	0.68
*Pax4*	606.7	507.0	487.6	581.9	558.8	0.91
*Pax6*	942.0	599.2	621.2	518.4	693.8	0.86
*Isl1*	936.3	516.3	850.3	715.7	834.1	0.62
*Foxa2*	1896.9	1349.5	1304.2	1318.7	1506.6	0.90
*Insm1*	1358.5	488.6	986.8	1078.4	1141.3	0.43
*Neurod1*	603.8	210.9	263.8	288.1	385.2	0.55
*Neurog3*	176.9	192.3	192.0	203.6	190.8	1.01
*Pcsk2*	4580.8	5429.3	5624.6	3924.2	4709.9	1.15
*Slc2a2*	478.5	499.2	463.8	1754.5	898.9	0.56
*Gck*	803.2	550.6	788.4	716.6	769.4	0.72
*Abcc8*	154.7	160.3	168.0	174.6	165.8	0.97
*Kcnj11*	815.8	644.0	1171.2	933.1	973.4	0.66
*Ins1*	28131.6	16852.5	27736.0	27214.7	27694.1	0.61
*Ins1*	19939.3	8937.0	18275.3	20041.0	19418.5	0.46
*Ins2*	14771.3	15525.9	15461.2	14919. 7	15050.7	1.03
*Sst*	206.1	206.4	211.3	191.4	203.0	1.02
*Ppy*	810.5	559.8	524.1	548.7	627.8	0.89
*Iapp*	8291.0	7443.6	9641.2	8110.5	8680.9	0.86

* Raw values of expression intensities measured by Affymetrix arrays. **Mean value of C4-LP, C4-HP, and Pr-LP. ***Ratio of Pr-HP to mean value.

There was no significant difference in the expression of the insulin genes (*Ins1* and *Ins2*) as assessed by the DNA microarray analysis. However, because the expression levels of the *Ins1* and *Ins2* genes were dramatically higher than those of the other genes, their signal intensities might have exceeded the linear range of detection. Therefore, we analyzed the expression of the *Ins1* and *Ins2* genes by quantitative RT-PCR. We found that the level of *Ins2* gene expression was not significantly different among the four groups, but that of *Ins1* was much lower in the Pr-HP cells than in the responder cells (**Figure 3E and 3F**). Thus, the *Ins1* gene can be considered a responder gene.

### Expression of a cell-surface protein, CD24

**Figure 4:**
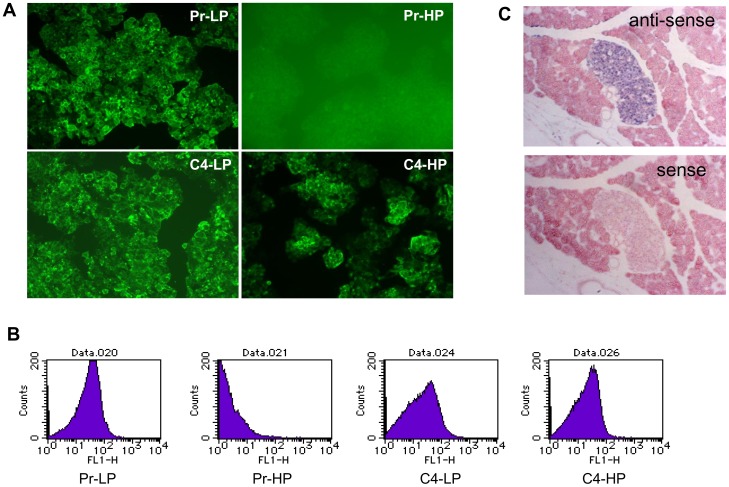
CD24 expression in MIN6 cells and analysis of *Hepacam2* expression by *in situ* hybridization. Immunocytochemical analysis (A) and flow cytometric analysis (B) of Pr-LP, Pr-HP, C4-LP, and C4-HP MIN6 cells with an anti-CD24 antibody. CD24 was not detected on the surface of Pr-HP cells. *In situ* hybridization analysis of *Hepacam2* expression in pancreatic islets (C). Pancreatic sections were hybridized with DIG-labeled anti-sense and sense RNA probes for *Hepacam2* transcripts (see **Materials and Methods**).

The DNA microarray data showed that *Cd24a* was expressed in the Pr-LP, C4-LP, and C4-HP cells, but not in the Pr-HP cells (**Figure 3G**). In addition, immunocytochemical analysis detected CD24 on the surface of Pr-LP, C4-LP, and C4-HP cells, but not on Pr-HP cells (**Figure 4A**). Flow-cytometric analysis also showed that CD24 was expressed similarly in Pr-LP, C4-LP, and C4-HP cells, but its expression was very low or lost in Pr-HP cells (**Figure 4B**). Interestingly, the *Cd24a* gene is expressed in the embryonic endocrine pancreas (E14.5), as shown by *in situ* hybridization (see http://www.genepaint.org/Frameset.html).

### Analysis of the *Hepacam2* gene

*Hepacam2* was highly expressed in the responder MIN6 cells, but not in the non-responder ones (**Figure 3H**). It encodes a putative membrane-anchored protein. *In situ* hybridization analysis showed that *Hepacam2* expression was restricted to the islets in the pancreas (**Figure 4C**). *Hepacam2* is also expressed in the embryonic pancreas (E14.5) [12].

### Methylated genes

**Figure 5:**
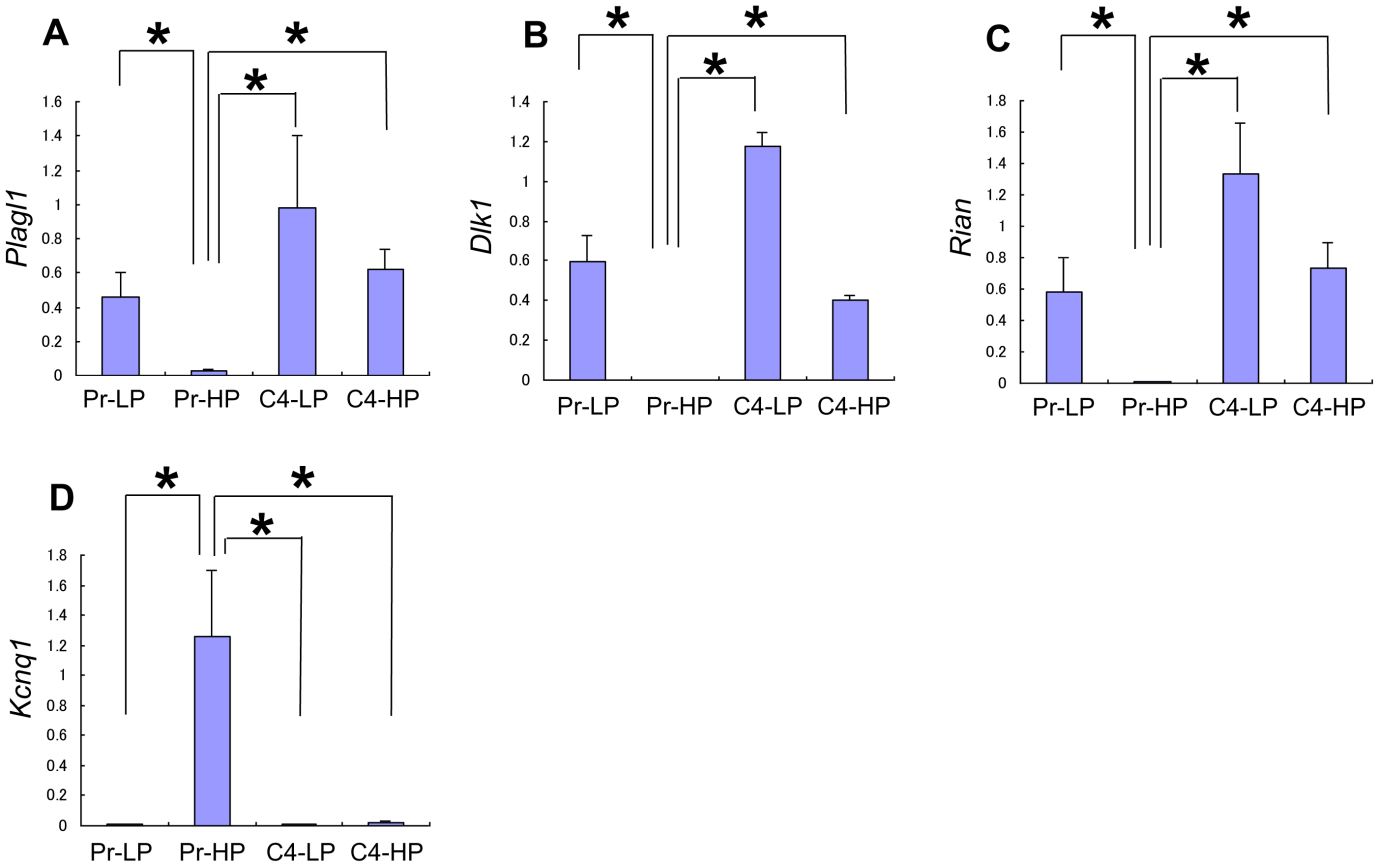
Quantitative RT-PCR analysis of imprinted genes. Expression of imprinted genes, *Rian* (A), *Plagl1* (B), *Dlk1* (C), and *Kcnq1* (D) in Pr-LP, Pr-HP, C4-LP, and C4-HP MIN6 cells. *Plagl1*, *Dlk1*, and *Rian* were confirmed to be responder genes, whereas *Kcnq1* was a non-responder gene. Values are means ± SD and n = 4–5. *P<0.05.

Interestingly, we found several imprinted genes among the differentially expressed genes. The responder genes included *Plagl1* (**Figure 5A**), *Dlk1* (**Figure 5B**), *Meg3*, *Nnat*, *Ndn*, *Mirg*, *Peg3*, and *Th*. *Cdkn1c* was a non-responder gene. Because only about 150 verified imprinted genes are known (http://igc.otago.ac.nz/1101Summary-table.pdf), the percentage of imprinted genes among the differentially expressed genes in our analysis seemed very high, especially for the responder genes. Therefore, we further investigated the expression of imprinted genes. *Dlk1*, *Meg3*, and *Mirg* are located in the well-known *Dlk1*-*Gtl1* imprinted gene cluster [13], [14]. An analysis of the expression patterns of other imprinted genes in the *Dlk1*-*Gtl1* locus indicated that *Rian* (**Figure 5C**) and *Rtl1* (data not shown) are also responder genes.

Recent reports have shown that variants in *KCNQ1* are associated with susceptibility to human type 2 diabetes mellitus [15], [16]. Interestingly, *Kcnq1* was reported to be an imprinted gene [13], [14]. Our DNA microarray analysis showed that the *Kcnq1* expression in Pr-HP cells was 2.2-fold higher than in responder cells. Therefore, we analyzed the expression of *Kcnq1* by quantitative RT-PCR. As shown in **Figure 5D**, *Kcnq1* was a typical non-responder gene.

**Figure 6:**
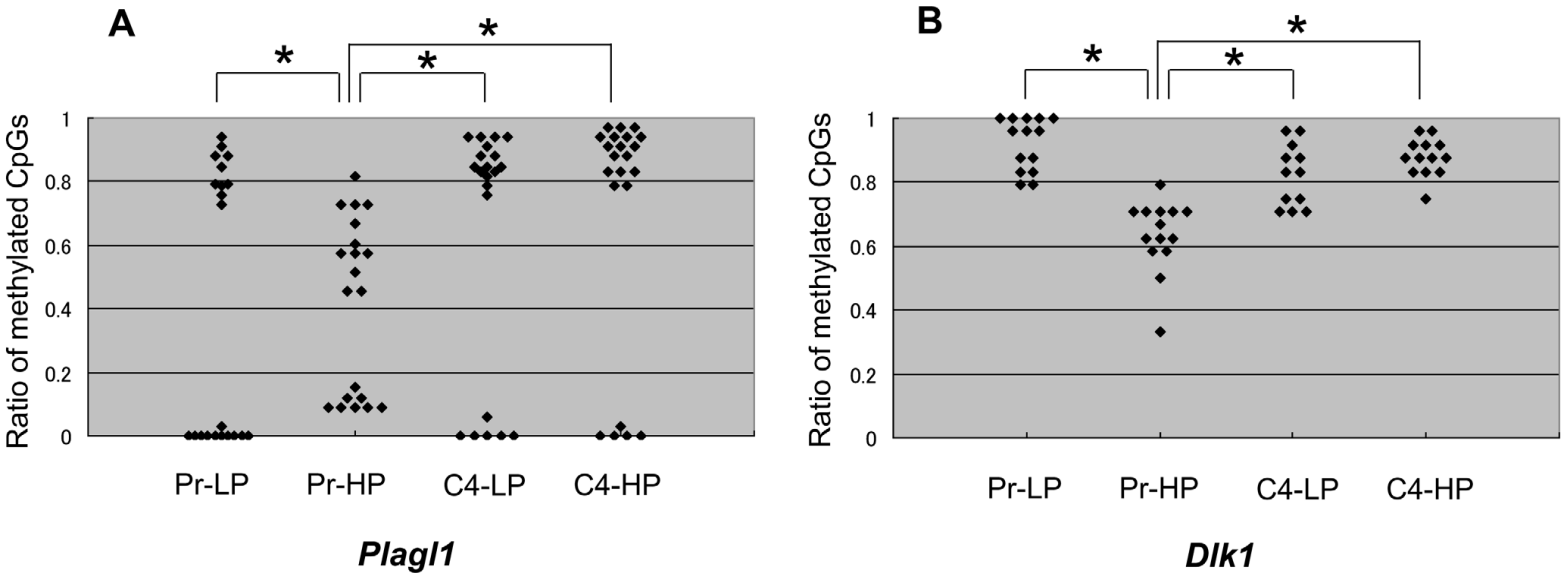
Analysis of CpG methylation of *Plagl1* and *Dlk1*. The ratio of methylated CpGs of each allele from Pr-LP, Pr-HP, C4-LP, and C4-HP MIN6 cells, in the *Plagl1* gene region (A) and the *Dlk1* gene region (B), by bisulfite sequencing. In the *Plagl1* gene locus, the pattern of methylation of CpG islands was quantitatively different between the responder MIN6 cells (Pr-LP, C4-LP, and C4-HP) and the non-responder cells (Pr-HP). In responder cells, one allele was either almost completely unmethylated and the other was almost completely methylated, whereas in the Pr-HP cells, both alleles were randomly methylated. In contrast, the *Dlk1* locus was significantly less methylated in Pr-HP cells than in the responder MIN6 cells. Values are means ± SD and n = 8-10. *P<0.

Genomic imprinting is an epigenetic form of gene regulation that involves differential DNA methylation of the paternal and maternal alleles of a gene. Such methylation is inherited in a parent-of-origin-specific manner. We investigated the methylation status of the known regulatory regions of the *Plagl1* and *Dlk1* genes, which were highly expressed in the responder MIN6 cells, but hardly at all in the non-responder cells (**Figure 5B and 5C**). Bisulfite methylation analysis revealed that in the *Plagl1* gene locus, the pattern of methylation of CpG islands was quantitatively different between the responder MIN6 cells (Pr-LP, C4-LP, and C4-HP) and the non-responder cells (Pr-HP) (**Figure 6A**). In the responder cells, one allele was almost completely unmethylated and the other almost completely methylated, whereas in the Pr-HP cells, both alleles appeared to be methylated at random. In contrast, the *Dlk1* locus was significantly less methylated in Pr-HP cells than in the responder cells (**Figure 6B**). Thus, the methylation levels of the CpG islands of imprinted genes differed between the responder and non-responder MIN6 cells, but the pattern of methylation differed according to the gene locus.

## Discussion

Although MIN6 cells are widely used as a model of pancreatic β cells, the GSIS is gradually lost with long-term culture. We previously isolated a subclone, MIN6 clone 4, from the parental MIN6 cells. This subclone showed stable GSIS even after long-term culture. To look for candidate genes for the maintenance of GSIS, we decided to analyze the gene expression patterns of Pr-LP, Pr-HP, C4-LP, and C4-HP cells. Our DNA microarray analysis extracted 60 differentially expressed responder genes (with consistently higher expression in Pr-LP, C4-LP, and C4-HP cells) and 62 non-responder genes (higher expression in the Pr-HP cells) (**Table S2**). Genes differentially expressed among Pr-LP, C4-LP, and C4-HP cells are also listed (Table S3, S4, S5). Among these genes, *Tmem59l* is also one of the responder genes (Table 1). As shown in Figure S1E, *Tmem59l* was highly expressed in the responder cells, but not in the non-responder cells. Its expression was higher in C4-LP and C4-HP cells than in Pr-LP cells. Further functional analysis is needed to know the roles of these differentially expressed genes in β cells.

Some of the responder genes have been implicated in the function or development of pancreatic β cells. One responder gene, *Chrebp* (official name: *Mlxipl*), encodes a transcription factor called carbohydrate-responsive element binding protein (ChREBP), and its expression increases in response to extracellular glucose in β cells [17]. It is also involved in the differentiation [18] and proliferation of β cells [19]. *Chrebp* was also reported to be expressed in the embryonic pancreas and to be a target of FOXA1 and FOXA2, which are crucial for the mature β-cell phenotype [20]. Our data suggested that *Chrebp* is also important for the physiological function of β cells.

Genes known to be involved in exocytosis were also found among the responder genes. *Syt7* is a member of the synaptotagmin family, which is crucial for the exocytosis of vesicles [21]. Its protein product, Synaptotagmin VII, participates in the calcium-dependent insulin release from pancreatic β cells [22], [23], and the ablation of *Syt7* impairs GSIS [24], [25]. Complexin II, encoded by *Cplx2*, is a cytoplasmic pre-synaptic protein believed to regulate neurotransmitter release from pre-synaptic terminals [26]. In our results, the Complexin II level appeared to be high in pancreatic β cells. Because *Cplx2* is a homologue of *Cplx1*, which is implicated in insulin secretion [27], [28], *Cplx2* may also play a role in insulin secretion. *Scgn,* which encodes a calcium-binding protein, is highly expressed in the pancreatic islets [29] and is also involved in the regulation of insulin secretion [30].

The Pr-HP MIN6 cells expressed some pancreas-related genes that are not normally expressed in mature β cells. For example, *Car2*, which encodes carbonic anhydrase 2, is expressed in duct cells and pancreatic α cells [31]. *Gcg*, encoding preproglucagon, is expressed in pancreatic α cells. *cMaf* (official name: *Maf*) encodes a transcription factor that belongs to the large Maf family, and the *cMaf* mRNA is detected in the embryonic pancreas [32], but it is not expressed in adult pancreatic β cells. *cMaf* was also reported to be expressed in pancreatic α cells and to have a role in their differentiation and maintenance [33]–[35].

The *Ins2* gene was expressed at high levels in all four MIN6 cell groups, whereas the *Ins1* gene expression was high in responder cells, but extremely low in Pr-HP cells (**Figure 3E**). *Ins1* and *Ins2* are regulated differently [36]–[39] and are under the control of different enhancer-promoter regions. The human insulin gene (*INS*) and the mouse *Ins2* gene have two introns, but the mouse *Ins1* gene lacks the second intron. Thus, the mouse *Ins2* gene appears more like the human insulin gene. Compensatory responses were reported in mice carrying a null mutation for *Ins1* or *Ins2* [40]. Therefore, the downregulation of *Ins1* might not account for the dysregulation of GSIS in Pr-HP cells. In pancreatic β-cell-specific *Neurod1* knockout mice, the *Ins1* gene expression is almost absent, whereas the *Ins2* gene expression is not much affected [41]. *Neurod1* expression in Pr-HP cells was approximately half that in the responder cells (**Table 3**), which may partly explain the low level of *Ins1* expression in the Pr-HP cells.

Our DNA microarray analysis identified *Cd24a* as a responder gene (**Figure 3G**). Immunohistochemical analysis confirmed that CD24 was expressed on the surface of Pr-LP cells and MIN6 clone 4 cells, but was low or absent in Pr-HP cells. Similarly, Cram *et al.* showed that the *Cd24* gene is expressed at higher levels in RIN-A12 insulinoma cells, which produce a larger amount of insulin, than in RIN-5AH insulinoma cells, which are low insulin producers [42]. Our present results may suggest that CD24 is a useful marker for β-cell lines that retain their differentiated phenotypes.

The *Hepacam2* gene was identified as a responder gene (**Figure 3H**). *Hepacacm2* encodes a putative membrane-anchored protein and was reported to be expressed in pancreatic endocrine cells, including β cells [43]. Our *in situ* hybridization analysis confirmed this (**Figure 4C**). It should be interesting to examine the possible roles of *Hepacam2* in the regulation of GSIS in β cells.

The present study also found a set of imprinted genes among the responder genes. The majority of known imprinted genes in mammals have roles in the control of embryonic growth and development, including development of the placenta [44]. Others are involved in post-natal development, with roles in suckling behavior and metabolism [44]. The imprinted responder genes *Plagl1*, *Dlk1*, and *Nnat* have been implicated in pancreatic β-cell function [45]–[51]. *Plagl1*, also called *Zac1*, was first isolated as a candidate gene for transient neonatal diabetes (TNDM) [45], which is a rare inherited diabetic syndrome apparent in the first weeks of human life and again during early adulthood. *Plagl1* encodes a proapoptotic zinc finger protein. To investigate its pancreatic function, Ma *et al*. developed a high-copy transgenic mouse line, *TNDM29*, carrying the human *TNDM* locus, including the *Plagl1* gene [46]. *TNDM29* neonates display hyperglycemia, and older adults have impaired glucose tolerance. *Plagl1* overexpression in β-cell lines impairs insulin secretion [47]. Combined with our data, these findings suggest that β-cell function requires the careful regulation of *Plagl1* expression levels.

*Dlk1* is expressed in the embryonic pancreas and is gradually restricted to β cells during development [48]. Its protein, Dlk1, is secreted from islets and a β-cell line, and *Dlk1* overexpression in RINm5F cells attenuates their proliferation [49].

*Nnat* expression was first discovered in the brain and is regulated by *Neurod1*; its suppression by siRNA represses the GSIS by β cells [50]. A recent report also revealed that *Nnat* is involved in the insulin secretory mechanism and in apoptosis [51]. Thus, the low expression of *Nnat* may partly account for the impairment of insulin secretion in Pr-HP cells.

Two other imprinted genes, *Meg3* (*Gtl2*) and *Mirg*, were identified as responder genes. These genes are located in the *Dlk1*-*Gtl2* gene cluster. Upon examination, we found that other genes in this locus, *Rtl1* and *Rian*, were also responder genes. The human *DLK1*-*MEG3* (*GTL2*) gene region was recently reported to affect susceptibility for type 1 diabetes [52]. Thus, this locus may contribute to the regulation of β-cell function.

In contrast to the above imprinted genes, *Kcnq1* was expressed at very low levels in the responder cells, but at high levels in the non-responder, Pr-HP cells. Recently *Kcnq1* was reported to be a candidate gene for type 2 diabetes susceptibility [13], [14]. *Kcnq1*-overexpressing MIN6 cells show a defect in insulin secretion in response to various secretagogues [53]. Thus, it is possible that high levels of *Kcnq1* expression lead to poor β-cell function.

DNA methylation is necessary for the proper expression of imprinted genes [13], [14] and is believed to represent the primary imprinting mark. Imprinted genes can be divided into two classes based on whether they are activated or repressed by the loss of DNA methylation. To clarify the mechanism for the regulation of imprinted genes in MIN6 cells, we analyzed the methylation status of the known regulatory loci of the *Plagl1* and *Dlk1* genes. One allele of the *Plagl1* gene locus was unmethylated and the other was highly methylated in responder cells, whereas both alleles were randomly methylated at various levels in Pr-HP cells (**Figure 6A**). *Plagl1* expression is reported to be downregulated by methylation [54]. Therefore, the random methylation of the *Plagl1* locus may account for its reduced expression in Pr-HP cells. On the other hand, both alleles of the *Dlk1* locus were highly methylated in responder cells, but were less methylated in non-responder cells (**Figure 6B**). An analysis of *Dnmt* ^−/−^ embryos indicated that *Dlk1* is positively regulated by DNA methylation [55]. Therefore, the reduced methylation of the *Dlk1* locus may account for the lower *Dlk1* gene expression in the Pr-HP cells.

Recently, an epigenetic mechanism was reported to be involved in the dysfunction of pancreatic β cells *in vivo*, resulting in the development of diabetes mellitus [56]–[60]. The imprinted genes found in the responder and non-responder genes may be important for the maintenance of β-cell functions. Further analysis of the genes identified by the global expression profiling of well-regulated and dysregulated MIN6 cells will help us to clarify the regulatory networks that control insulin secretion.

## Supporting Information

Figure S1 **Quantitative RT-PCR analysis.** Expression of the genes of interest, *Chrebp* (A), *Syt7* (B), *Cplx2* (C), *Scgn* (D), and *Tmem59l* (E) in Pr-LP, Pr-HP, C4-LP, and C4-HP MIN6 cells was examined by quantitative RT-PCR. These genes were confirmed to be responder genes. n = 4–5. Values are means ± SD. *P<0.05.
(TIF)

Table S1 **PCR primers used in the present study.**
(PDF)

Table S2 **Genes differentially expressed between responder and non-responder MIN6 cells.**
(PDF)

Table S3 **Genes differentially expressed between Pr-LP and clone 4 MIN6 cells.**
(PDF)

Table S4 **Genes differentially expressed between Pr-LP and C4-LP MIN6 cells.**
(PDF)

Table S5 **Genes differentially expressed between C4-LP and C4-HP MIN6 cells.**
(PDF)

## References

[1] Miyazaki J, Araki K, Yamato E, Ikegami H, Asano T, et al (1990) Establishment of a pancreatic beta cell line that retains glucose-inducible insulin secretion: special reference to expression of glucose transporter isoforms. Endocrinology 127: 126–132. 2163307 10.1210/endo-127-1-126

[2] Kayo T, Sawada Y, Suzuki Y, Suda M, Tanaka S, et al (1996) Proprotein-processing endoprotease furin decreases regulated secretory pathway-specific proteins in the pancreatic beta cell line MIN6. J Biol Chem 271: 10731–10737. 8631882 10.1074/jbc.271.18.10731

[3] Sawada Y, Zhang B, Okajima F, Izumi T, Takeuchi T (2001) PTHrP increases pancreatic beta-cell-specific functions in well-differentiated cells. Mol Cell Endocrinol 182: 265–275. 11514060 10.1016/s0303-7207(01)00482-8

[4] O'Driscoll L, Gammell P, McKiernan E, Ryan E, Jeppesen PB, et al (2006) Phenotypic and global gene expression profile changes between low passage and high passage MIN-6 cells. J Endocrinol 191: 665–676. 17170223 10.1677/joe.1.06894

[5] Minami K, Yano H, Miki T, Nagashima K, Wang CZ, et al (2000) Insulin secretion and differential gene expression in glucose-responsive and -unresponsive MIN6 sublines. Am J Physiol Endocrinol Metab 279: E773–781. 11001758 10.1152/ajpendo.2000.279.4.E773

[6] Lilla V, Webb G, Rickenbach K, Maturana A, Steiner DF, et al (2003) Differential gene expression in well-regulated and dysregulated pancreatic beta-cell (MIN6) sublines. Endocrinology 144: 1368–1379. 12639920 10.1210/en.2002-220916

[7] Dowling P, O'Driscoll L, O'Sullivan F, Dowd A, Henry M, et al (2006) Proteomic screening of glucose-responsive and glucose non-responsive MIN-6 beta cells reveals differential expression of proteins involved in protein folding, secretion and oxidative stress. Proteomics 6: 6578–6587. 17163442 10.1002/pmic.200600298

[8] Dowling P, Shields W, Rani S, Meleady P, Henry M, et al (2008) Proteomic analysis of conditioned media from glucose responsive and glucose non-responsive phenotypes reveals a panel of secreted proteins associated with beta cell dysfunction. Electrophoresis 29: 4141–4149. 18924105 10.1002/elps.200800152

[9] Cheng K, Delghingaro-Augusto V, Nolan CJ, Turner N, Hallahan N, et al (2012) High passage MIN6 cells have impaired insulin secretion with impaired glucose and lipid oxidation. PLoS One 7: e40868. 22808281 10.1371/journal.pone.0040868 PMC3396628

[10] Hiura H, Obata Y, Komiyama J, Shirai M, Kono T (2006) Oocyte growth-dependent progression of maternal imprinting in mice. Genes to Cells 11: 353–361. 16611239 10.1111/j.1365-2443.2006.00943.x

[11] Takada S, Paulsen M, Tevendale M, Tsai CE, Kelsey G, et al (2002) Epigenetic analysis of the Dlk1-Gtl2 imprinted domain on mouse chromosome 12: implications for imprinting control from comparison with Igf2-H19. Hum Mol Genet 11: 77–86. 11773001 10.1093/hmg/11.1.77

[12] Hoffman BG, Zavaglia B, Witzsche J, Ruiz de Algara T, Beach M, et al (2008) Identification of transcripts with enriched expression in the developing and adult pancreas. Genome Biol 9: R99. 18554416 10.1186/gb-2008-9-6-r99 PMC2481431

[13] Morison IM, Ramsay JP, Spencer HG (2005) A census of mammalian imprinting. Trends Genet 21: 457–465. 15990197 10.1016/j.tig.2005.06.008

[14] Edwards CA, Ferguson-Smith AC (2007) Mechanisms regulating imprinted genes in clusters. Curr Opin Cell Biol 19: 281–289. 17467259 10.1016/j.ceb.2007.04.013

[15] Yasuda K, Miyake K, Horikawa Y, Hara K, Osawa H, et al (2008) Variants in KCNQ1 are associated with susceptibility to type 2 diabetes mellitus. Nat Genet 40: 1092–1097. 18711367 10.1038/ng.207

[16] Unoki H, Takahashi A, Kawaguchi T, Hara K, Horikoshi M, et al (2008) SNPs in KCNQ1 are associated with susceptibility to type 2 diabetes in East Asian and European populations. Nat Genet 40: 1098–1102. 18711366 10.1038/ng.208

[17] Leclerc I, Rutter GA, Meur G, Noordeen N (2012) Roles of Ca^2+^ions in the control of ChREBP nuclear translocation. J Endocrinol 213: 115–122. 22402852 10.1530/JOE-11-0480

[18] Metukuri MR, Zhang P, Basantani MK, Chin C, Stamateris RE, et al (2012) ChREBP mediates glucose-stimulated pancreatic β-cell proliferation. Diabetes 61: 2004–2015. 22586588 10.2337/db11-0802 PMC3402328

[19] Soggia A, Flosseau K, Ravassard P, Szinnai G, Scharfmann R, et al (2012) Activation of the transcription factor carbohydrate-responsive element-binding protein by glucose leads to increased pancreatic beta cell differentiation in rats. Diabetologia 55: 2713–2722. 22760788 10.1007/s00125-012-2623-0 PMC3433661

[20] Gao N, Le Lay J, Qin W, Doliba N, Schug J, et al (2010) Foxa1 and Foxa2 maintain the metabolic and secretory features of the mature beta-cell. Mol Endocrinol 24: 1594–1604. 20534694 10.1210/me.2009-0513 PMC2940470

[21] Gauthier BR, Wollheim CB (2008) Synaptotagmins bind calcium to release insulin. Am J Physiol Endocrinol Metab 295: E1279–1286. 18713958 10.1152/ajpendo.90568.2008

[22] Gao Z, Reavey-Cantwell J, Young RA, Jegier P, Wolf BA (2000) Synaptotagmin III/VII isoforms mediate Ca^2+^-induced insulin secretion in pancreatic islet beta-cells. J Biol Chem 275: 36079–36085. 10938083 10.1074/jbc.M004284200

[23] Gauthier BR, Duhamel DL, Iezzi M, Theander S, Saltel F, et al (2008) Synaptotagmin VII splice variants alpha, beta, and delta are expressed in pancreatic beta-cells and regulate insulin exocytosis. FASEB J 22: 194–206. 17709608 10.1096/fj.07-8333com

[24] Li Y, Wang P, Xu J, Gorelick F, Yamazaki H, et al (2007) Regulation of insulin secretion and GLUT4 trafficking by the calcium sensor synaptotagmin VII. Biochem Biophys Res Commun 362: 658–664. 17720139 10.1016/j.bbrc.2007.08.023 PMC2194288

[25] Gustavsson N, Lao Y, Maximov A, Chuang JC, Kostromina E, et al (2008) Impaired insulin secretion and glucose intolerance in synaptotagmin-7 null mutant mice. Proc Natl Acad Sci USA 105: 3992–3997. 18308938 10.1073/pnas.0711700105 PMC2268794

[26] Kataoka M, Sekiguchi M, Takahashi M (2009) Identification of a minimal segment of complexin II essential for preferential distribution in axons. J Neurochem 108: 1109–1115. 19141077 10.1111/j.1471-4159.2009.05874.x

[27] Abderrahmani A, Niederhauser G, Plaisance V, Roehrich ME, Lenain V, et al (2004) Complexin I regulates glucose-induced secretion in pancreatic beta-cells. J Cell Sci 117: 2239–2247. 15126625 10.1242/jcs.01041

[28] Maj M, Gartner W, Ilhan A, Neziri D, Attems J, et al (2010) Expression of TAU in insulin-secreting cells and its interaction with the calcium-binding protein secretagogin. J Endocrinol 205: 25–36. 20061514 10.1677/JOE-09-0341

[29] Wagner L, Oliyarnyk O, Gartner W, Nowotny P, Groeger M, et al (2000) Cloning and expression of secretagogin, a novel neuroendocrine- and pancreatic islet of Langerhans-specific Ca^2+^-binding protein. J Biol Chem 275: 24740–24751. 10811645 10.1074/jbc.M001974200

[30] Gartner W, Vila G, Daneva T, Nabokikh A, Koc-Saral F, et al (2007) New functional aspects of the neuroendocrine marker secretagogin based on the characterization of its rat homolog. Am J Physiol Endocrinol Metab 293: E347–354. 17426113 10.1152/ajpendo.00055.2007

[31] Inada A, Nienaber C, Fonseca S, Bonner-Weir S (2006) Timing and expression pattern of carbonic anhydrase II in pancreas. Dev Dyn 235: 1571–1577. 16586439 10.1002/dvdy.20754

[32] Nishimura W, Kondo T, Salameh T, El Khattabi I, Dodge R, et al (2006) A switch from MafB to MafA expression accompanies differentiation to pancreatic beta-cells. Dev Biol 293: 526–539. 16580660 10.1016/j.ydbio.2006.02.028 PMC2390934

[33] Gosmain Y, Cheyssac C, Heddad Masson M, Dibner C, Philippe J (2011) Glucagon gene expression in the endocrine pancreas: the role of the transcription factor Pax6 in alpha-cell differentiation, glucagon biosynthesis and secretion. Diabetes Obes Metab 13 Suppl 131–38. 21824254 10.1111/j.1463-1326.2011.01445.x

[34] Katz LS, Gosmain Y, Marthinet E, Philippe J (2009) Pax6 regulates the proglucagon processing enzyme PC2 and its chaperone 7B2. Mol Cell Biol 29: 2322–2334. 19223471 10.1128/MCB.01543-08 PMC2663301

[35] Gosmain Y, Avril I, Mamin A, Philippe J (2007) Pax-6 and c-Maf functionally interact with the alpha-cell-specific DNA element G1 in vivo to promote glucagon gene expression. J Biol Chem 282: 35024–35034. 17901057 10.1074/jbc.M702795200

[36] Meur G, Qian Q, da Silva Xavier G, Pullen TJ, Tsuboi T, et al (2011) Nucleo-cytosolic shuttling of FoxO1 directly regulates mouse Ins2 but not Ins1 gene expression in pancreatic beta cells (MIN6). J Biol Chem 286: 13647–13656. 21335550 10.1074/jbc.M110.204248 PMC3075709

[37] Bengtsson M, Stahlberg A, Rorsman P, Kubista M (2005) Gene expression profiling in single cells from the pancreatic islets of Langerhans reveals lognormal distribution of mRNA levels. Genome Res 15: 1388–1392. 16204192 10.1101/gr.3820805 PMC1240081

[38] Ling Z, Heimberg H, Foriers A, Schuit F, Pipeleers D (1998) Differential expression of rat insulin I and II messenger ribonucleic acid after prolonged exposure of islet beta-cells to elevated glucose levels. Endocrinology 139: 491–495. 9449616 10.1210/endo.139.2.5749

[39] Giddings SJ, Carnaghi LR, Fischer LJ, Miller CP (1991) Differential regulation of rat insulin I and II messenger RNA synthesis: effects of fasting and cyproheptadine. Mol Endocrinol 5: 549–554. 1922087 10.1210/mend-5-4-549

[40] Leroux L, Desbois P, Lamotte L, Duvillie B, Cordonnier N, et al (2001) Compensatory responses in mice carrying a null mutation for Ins1 or Ins2. Diabetes 50 Suppl 1S150–153. 11272179 10.2337/diabetes.50.2007.s150

[41] Gu C, Stein GH, Pan N, Goebbels S, Hornberg H, et al (2010) Pancreatic beta cells require NeuroD to achieve and maintain functional maturity. Cell Metabolism 11: 298–310. 20374962 10.1016/j.cmet.2010.03.006 PMC2855640

[42] Cram DS, McIntosh A, Oxbrow L, Johnston AM, DeAizpurua HJ (1999) Differential mRNA display analysis of two related but functionally distinct rat insulinoma (RIN) cell lines: identification of CD24 and its expression in the developing pancreas. Differentiation 64: 237–246. 10365441 10.1046/j.1432-0436.1999.6440237.x

[43] Hald J, Galbo T, Rescan C, Radzikowski L, Sprinkel AE, et al (2012) Pancreatic islet and progenitor cell surface markers with cell sorting potential. Diabetologia 55: 154–165. 21947380 10.1007/s00125-011-2295-1

[44] Tycko B, Morison IM (2002) Physiological functions of imprinted genes. J Cell Physiol 192: 245–258. 12124770 10.1002/jcp.10129

[45] Kamiya M, Judson H, Okazaki Y, Kusakabe M, Muramatsu M, et al (2000) The cell cycle control gene ZAC/PLAGL1 is imprinted--a strong candidate gene for transient neonatal diabetes. Hum Mol Genet 9: 453–460. 10655556 10.1093/hmg/9.3.453

[46] Ma D, Shield JP, Dean W, Leclerc I, Knauf C, et al (2004) Impaired glucose homeostasis in transgenic mice expressing the human transient neonatal diabetes mellitus locus, TNDM. J Clin Invest 114: 339–348. 15286800 10.1172/JCI19876 PMC484972

[47] Hoffmann A, Spengler D (2012) Transient neonatal diabetes mellitus gene Zac1 impairs insulin secretion in mice through Rasgrf1. Mol Cell Biol 32: 2549–2560. 22547676 10.1128/MCB.06637-11 PMC3434484

[48] Carlsson C, Tornehave D, Lindberg K, Galante P, Billestrup N, et al (1997) Growth hormone and prolactin stimulate the expression of rat preadipocyte factor-1/delta-like protein in pancreatic islets: molecular cloning and expression pattern during development and growth of the endocrine pancreas. Endocrinology 138: 3940–3948. 9275085 10.1210/endo.138.9.5408

[49] Friedrichsen BN, Carlsson C, Moldrup A, Michelsen B, Jensen CH, et al (2003) Expression, biosynthesis and release of preadipocyte factor-1/delta-like protein/fetal antigen-1 in pancreatic beta-cells: possible physiological implications. J Endocrinol 176: 257–266. 12553874 10.1677/joe.0.1760257

[50] Chu K, Tsai MJ (2005) Neuronatin, a downstream target of BETA2/NeuroD1 in the pancreas, is involved in glucose-mediated insulin secretion. Diabetes 54: 1064–1073. 15793245 10.2337/diabetes.54.4.1064 PMC1197706

[51] Joe MK, Lee HJ, Suh YH, Han KL, Lim JH, et al (2008) Crucial roles of neuronatin in insulin secretion and high glucose-induced apoptosis in pancreatic beta-cells. Cell Signal 20: 907–915. 18289831 10.1016/j.cellsig.2008.01.005

[52] Wallace C, Smyth DJ, Maisuria-Armer M, Walker NM, Todd JA, et al (2010) The imprinted DLK1-MEG3 gene region on chromosome 14q32.2 alters susceptibility to type 1 diabetes. Nat Genet 42: 68–71. 19966805 10.1038/ng.493 PMC2820243

[53] Yamagata K, Senokuchi T, Lu M, Takemoto M, Fazlul Karim M, et al (2011) Voltage-gated K+channel KCNQ1 regulates insulin secretion in MIN6 beta-cell line. Biochem Biophys Res Commun 407: 620–625. 21426901 10.1016/j.bbrc.2011.03.083

[54] Varrault A, Bilanges B, Mackay DJ, Basyuk E, Ahr B, et al (2001) Characterization of the methylation-sensitive promoter of the imprinted ZAC gene supports its role in transient neonatal diabetes mellitus. J Biol Chem 276: 18653–18656. 11297535 10.1074/jbc.C100095200

[55] Schmidt JV, Matteson PG, Jones BK, Guan XJ, Tilghman SM (2000) The Dlk1 and Gtl2 genes are linked and reciprocally imprinted. Genes Dev 14: 1997–2002. 10950864 PMC316857

[56] Yang BT, Dayeh TA, Volkov PA, Kirkpatrick CL, Malmgren S, et al (2012) Increased DNA Methylation and Decreased Expression of PDX-1 in Pancreatic Islets from Patients with Type 2 Diabetes. Mol Endocrinol 26: 1203–1212. 22570331 10.1210/me.2012-1004 PMC5416998

[57] Volkmar M, Dedeurwaerder S, Cunha DA, Ndlovu MN, Defrance M, et al (2012) DNA methylation profiling identifies epigenetic dysregulation in pancreatic islets from type 2 diabetic patients. EMBO J 31: 1405–1426. 22293752 10.1038/emboj.2011.503 PMC3321176

[58] Yang BT, Dayeh TA, Kirkpatrick CL, Taneera J, Kumar R, et al (2011) Insulin promoter DNA methylation correlates negatively with insulin gene expression and positively with HbA(1c) levels in human pancreatic islets. Diabetologia 54: 360–367. 21104225 10.1007/s00125-010-1967-6 PMC3017313

[59] Kuroda A, Rauch TA, Todorov I, Ku HT, Al-Abdullah IH, et al (2009) Insulin gene expression is regulated by DNA methylation. PLoS One 4: e6953. 19742322 10.1371/journal.pone.0006953 PMC2735004

[60] Park JH, Stoffers DA, Nicholls RD, Simmons RA (2008) Development of type 2 diabetes following intrauterine growth retardation in rats is associated with progressive epigenetic silencing of Pdx1. J Clin Invest 118: 2316–2324. 18464933 10.1172/JCI33655 PMC2373422

