# Supplementary figures and images for: Microarray Analysis of Novel Candidate Genes Responsible for Glucose-Stimulated Insulin Secretion in Mouse Pancreatic β Cell Line MIN6
Source: PLoS One. 2013 Apr 3;8(4):e61211. doi: 10.1371/journal.pone.0061211 (PMC3616144; doi:10.1371/journal.pone.0061211)

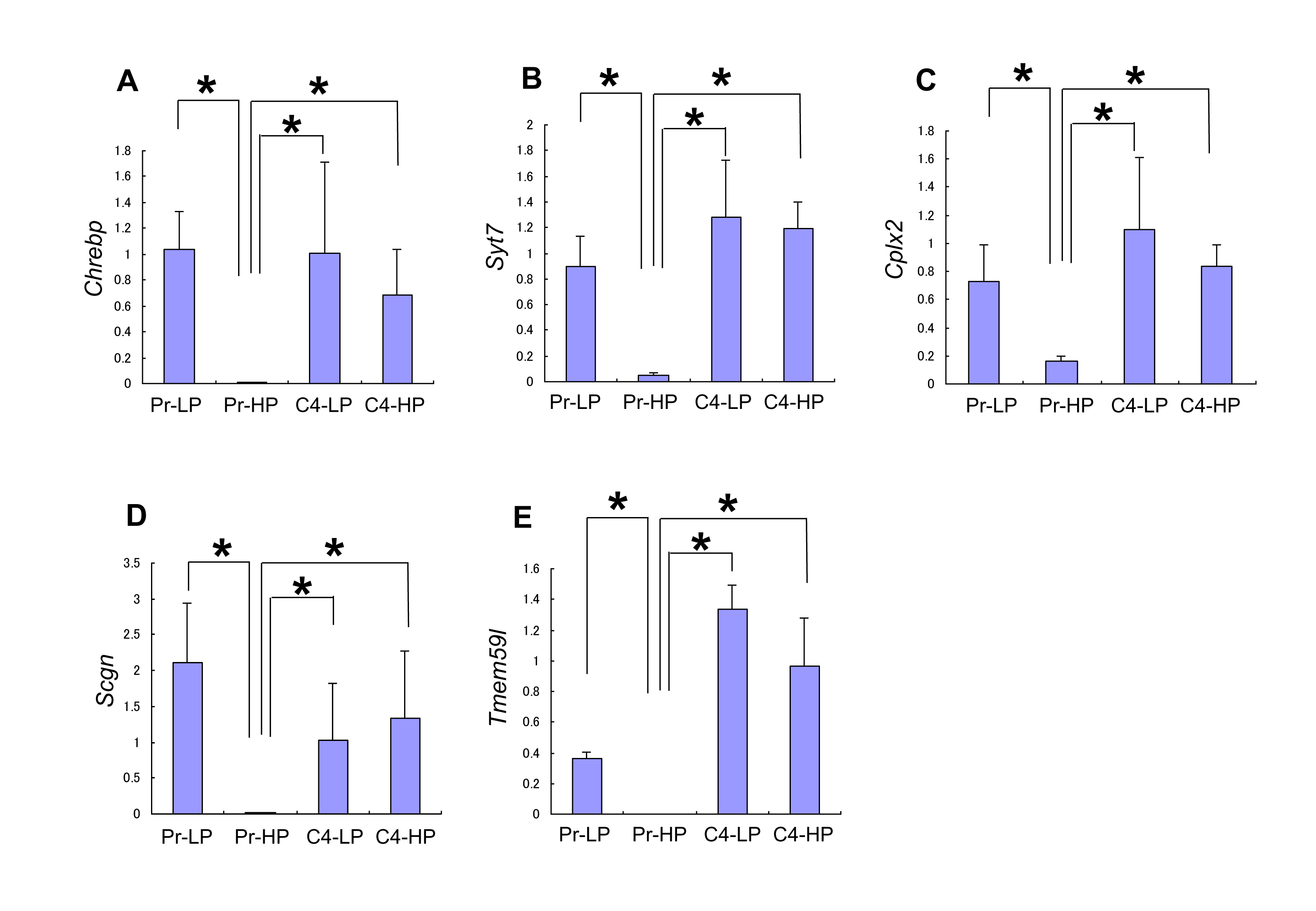

Supplement: Figure S1 — Quantitative RT-PCR analysis. Expression of the genes of interest, Chrebp (A), Syt7 (B), Cplx2 (C), Scgn (D), and Tmem59l (E) in Pr-LP, Pr-HP, C4-LP, and C4-HP MIN6 cells was examined by quantitative RT-PCR. These genes were confirmed to be responder genes. n = 4–5. Values are means ± SD. *P<0.05. (TIF) [file pone.0061211.s001.tif]
