# Supplementary material for: Microarray Analysis of Novel Candidate Genes Responsible for Glucose-Stimulated Insulin Secretion in Mouse Pancreatic β Cell Line MIN6
Source: PLoS One. 2013 Apr 3;8(4):e61211. doi: 10.1371/journal.pone.0061211 (PMC3616144; doi:10.1371/journal.pone.0061211)
Supplement: Table S1 — PCR primers used in the present study. (PDF) [file pone.0061211.s002.pdf]

Table S1. PCR primers used in the present study.

Gene	Forward primer (5' to 3')	Reverse primer (5' to 3')
Car2	GCAACCGGATGGATTGGCTG	CAGTTGTCCACCATCGCTTC
Maf	ATCCGACTGAAGCAGAAGAG	TTAACAAGGTGGCTAGCTGG
Gcg	TTACTTTGTGGCTGGATTGCTT	AGTGGCGTTTGTCTTCATTCA
Mafa	TTCAGCAAGGAGGAGGTCAT	CCGCCAACTTCTCGTATTTC
Ins1	GCCCTTAGTGACCAGCTATAATCAG	GGGTAGGAAGTGCACCAACAG
Ins2	GCTTCTTCTACACACCCATGTC	AGCACTGATCTACAATGCCAC
Cd24a	ACCGAACATCTAGAGAGTCG	GCCTGAGTCTCTAACAGTAG
Hepacam2	AAGCTAGAAGGCAGACCAGA	GCCCATTGAATCTCACTCTG
Chrebp	TGCACAACTGGAAGTTCTGG	GGAGCTTGGAAACTTTCACC
Syt7	CTCGAAACCTCAAAGCCATG	AGCGATCATGTCCTTCCAGT
Cplx2	AAGAGCGCAAGGCGAAACAC	ATTTGAGCACTGTGTCCAGG
Scgn	TCTCCTCAAGGGCCTCATTTT	TGGATCACCAGGCGATAGG
Plagl1	CCCGAGAACGGGCTTGA	GCAGCAAGCACTAGACCATGAA
Dlk1	TTCTCTGGAAAGGACTGCCA	TCTTGTCGACGAATCCAGCT
Rian	AACAGATGCCTTCCTCACTG	TTTGTGAGCTCAGCAGTCCT
Kcnq1	CTGGATCAGTCCATTGGGAA	AGGAAGAGCTCAGGGTTGAT
Tmem59l	AGAGTGACTTCCTCAGTTGC	AGTAGGCTGGAGATTACAGC
Rpl32	CAATGTGTCCTCTAAGAACCGAAA	CCTGGCGTTGGGATTGG
