# Supplementary material for: Microarray Analysis of Novel Candidate Genes Responsible for Glucose-Stimulated Insulin Secretion in Mouse Pancreatic β Cell Line MIN6
Source: PLoS One. 2013 Apr 3;8(4):e61211. doi: 10.1371/journal.pone.0061211 (PMC3616144; doi:10.1371/journal.pone.0061211)
Supplement: Table S2 — Genes differentially expressed between responder and non-responder MIN6 cells. (PDF) [file pone.0061211.s003.pdf]

Table S2. Genes differentially expressed between responder and non-respoder MIN6 cells.

Probe*	Gene symbol	Pr-LP**	Pr-HP**	C4-LP**	C4-HP**	Responder mean***	Fold change****	Gene title
162573_at	*Tmem59l*	1382.48	3.77	4633.73	3903.26	3306.8	876.52	transmembrane protein 59-like
115141_at	*Mlxipl*	1289.8	< 1.0	400.25	559.47	750.01	> 750.01	MLX interacting protein-like
136724_at	*Scgn*	4857.91	10.27	937.44	2256.93	2686.64	261.60	secretagogin, EF-hand calcium binding protein
165402_f_at	*Tmed6*	72.29	< 1.0	141.78	108.62	107.68	> 107.68	transmembrane emp24 protein transport domain containing 6
166084_f_at	*Plagl1*	4760.66	113.97	4340.53	4734.19	4616.12	40.50	pleiomorphic adenoma gene-like 1
108813_at	*Hepacam2*	3166.87	73.53	2279.28	3058.82	2767.27	37.63	HEPACAM family member 2
101975_at	*Dlk1*	10836.9	323.7	13653	9169.26	11244.97	34.74	delta-like 1 homolog (Drosophila)
163814_r_at	*Plagl1*	404.02	14	517.93	494.88	472.77	33.78	pleiomorphic adenoma gene-like 1
115180_at	*Rps6kb1*	106.57	5.36	135.72	117	119.04	22.21	ribosomal protein S6 kinase, polypeptide 1
167047_r_at	*Syt7*	1375.05	65.93	1276.32	1342.03	1327.9	20.14	synaptotagmin VII
134115_at	*Slc29a4*	1514.41	78.87	1391.43	1682.69	1531.14	19.41	solute carrier family 29 (nucleoside transporters), member 4
100600_at	*Cd24a*	5664.5	282.53	5903.81	2859.73	4805.98	17.01	CD24a antigen
95550_at	*Rpgr*	103.54	7.27	110.1	143.31	119.31	16.42	retinitis pigmentosa GTPase regulator
160173_at	*Meg3*	1806.63	154.34	3021.36	2563.43	2463.4	15.96	maternally expressed 3
94852_at	*Glul*	1079.61	53.22	450.34	868.89	798.05	15.00	glutamate-ammonia ligase (glutamine synthetase)
92502_at	*Plagl1*	1827.41	143.67	2251.62	2323.89	2135.97	14.87	pleiomorphic adenoma gene-like 1
112870_at	*1700019D03Rik*	276.35	15.17	203.15	167.1	210.73	13.89	RIKEN cDNA 1700019D03 gene
107313_at	*Gm2115*	658.54	44.75	696.43	470.63	607.76	13.58	predicted gene 2115
138946_at	*Syt7*	845.06	68.94	800.04	776.73	805.6	11.69	synaptotagmin VII
97520_s_at	*Nnat*	8742.89	637.71	5415.87	7260.28	7113.34	11.15	neuronatin
92501_s_at	*Plagl1*	661.95	80.26	933.27	976.31	856.92	10.68	pleiomorphic adenoma gene-like 1

164148_at	*Meg3*	63.39	12.48	258.36	76.63	132.58	10.62	maternally expressed 3
101059_at	*Ndn*	319.41	94.57	1397.09	1183.73	966.54	10.22	necdin
101487_f_at	*Ly6e*	2310.75	250.92	3453.39	1776.05	2512.23	10.01	lymphocyte antigen 6 complex, locus E
169192_i_at	*Dlk1*	422.41	42.96	562.46	290.19	425.11	9.89	delta-like 1 homolog (Drosophila)
103942_at	*Gucy2c*	1148.21	217.57	2218.71	3054.82	2118.93	9.74	guanylate cyclase 2c
92665_f_at	*3830403N18Rik /// Xlr*	416.26	37.99	479.83	141.8	345.91	9.10	RIKEN cDNA 3830403N18 gene
104735_at	*Kctd12*	584.7	73.39	541.38	881.58	664.97	9.06	potassium channel tetramerisation domain containing 12
101851_at	*Cd200*	586.06	61.74	714.7	224.86	508.32	8.23	CD200 antigen
100690_at	*Th*	4282.22	477.77	4994.45	1843.84	3705.05	7.75	tyrosine hydroxylase
114644_at	*Amdhd2*	177.44	34.91	251.26	325.06	252.76	7.24	amidohydrolase domain containing 2
100772_g_at	*Blnk*	2164.57	414.8	4979.05	1727.94	2957.58	7.13	B-cell linker
136592_f_at	*Celsr2*	204.17	31.64	237.02	221.84	221.2	6.99	cadherin, EGF LAG seven-pass G-type receptor 2 (flamingo homolog, Drosophila)
106168_at	*Cplx2*	806.78	122.19	792.06	966.81	852.71	6.98	complexin 2
165753_at	*Tmod2*	751.02	77.06	360.97	477.58	530.15	6.88	tropomodulin 2
164137_at	*Cdhr1*	3040.65	288.52	965.41	1935.4	1980.31	6.86	cadherin-related family member 1
165756_at	*Ppp1r3d*	608.44	73.84	261.91	618.87	495.84	6.72	protein phosphatase 1, regulatory subunit 3D
97926_s_at	*Pparg*	121.73	21.38	208.11	92.27	140.67	6.58	peroxisome proliferator activated receptor gamma
166517_f_at	*Alcam*	341.37	42.46	137.28	356.71	278.07	6.55	activated leukocyte cell adhesion molecule
138453_at	*Unc80*	665.91	93.25	616.18	450.78	578.96	6.21	unc-80 homolog (C. elegans)
108096_at	*Epb4.1l4b*	2178.51	270.6	1110.42	1715.79	1667.52	6.16	erythrocyte protein band 4.1-like 4b
111046_r_at	*Akr1c14*	692.06	91.55	572.86	414.73	559.74	6.11	aldo-keto reductase family 1, member C14
105715_at	*A830039N20Rik*	216.76	48.32	402.66	238.62	285.8	5.91	RIKEN cDNA A830039N20 gene
112937_at	*Mirg*	193.5	41.59	278.57	246.87	240.41	5.78	miRNA containing gene
138087_at	*Fgf12*	493.39	73.32	321.4	428.88	414.59	5.65	fibroblast growth factor 12

162947_at	*Slc44a1*	261.52	39.14	213.81	187.67	218.22	5.57	solute carrier family 44, member 1
103052_r_at	*Nr2f2*	207.46	47.33	179.86	319.72	235.61	4.98	nuclear receptor subfamily 2, group F, member 2
95024_at	*Usp18*	659.66	103.29	358.41	523.56	513.93	4.98	ubiquitin specific peptidase 18
96143_at	*Epb4.1l4b*	743.84	138.42	465.19	737.47	648.43	4.68	erythrocyte protein band 4.1-like 4b
103238_at	*Wnt4*	346.88	76.99	387.88	336.29	357.85	4.65	wingless-related MMTV integration site 4
106933_at	*Phactr1*	290.55	60.68	278.58	277.51	282	4.65	phosphatase and actin regulator 1
167230_f_at	*Anxa4*	1407.66	238.27	1318.07	592.57	1106.3	4.64	annexin A4
113747_at	*Ptprn2*	4885.85	871.19	3693.4	3340.22	3966.46	4.55	protein tyrosine phosphatase, receptor type, N polypeptide 2
163880_at	*Uaca*	337.07	57.31	164.69	275.84	258.37	4.51	uveal autoantigen with coiled-coil domains and ankyrin repeats
110653_at	*Ino80d*	852.66	195.81	786.5	878.4	838.89	4.28	INO80 complex subunit D
106549_at	*Phactr1*	873.48	205.57	832.11	882.87	864.34	4.20	phosphatase and actin regulator 1
93390_g_at	*Prom1*	791.74	337.9	1757.55	1661.5	1404.04	4.16	prominin 1
162923_at	*Iqgap2*	352.02	89.41	337.84	432.83	369.49	4.13	IQ motif containing GTPase activating protein 2
99197_at	*Gc*	603.29	205.49	943.05	921.5	810.98	3.95	group specific component
164850_f_at	*Pvrl2*	988.78	187.68	705.8	487.88	726.48	3.87	poliovirus receptor-related 2
111016_at	*Tm4sf4*	2601.32	674.03	2985.65	1903.87	2496.24	3.70	transmembrane 4 superfamily member 4
92403_at	*St6galnac5*	764.14	210.24	883.75	643.86	763.48	3.63	ST6 (alpha-N-acetyl-neuraminyl-2,3-beta-galactosyl-1,3)-N-acetylgalactosaminide alpha-2,6-sialyltransferase 5
94429_at	*Eef1a2*	4857.48	1573.62	5991.6	6316.12	5671.21	3.60	eukaryotic translation elongation factor 1 alpha 2
101451_at	*Peg3*	3648.38	840.61	2717.96	2538.78	2962.66	3.52	paternally expressed 3
163413_at	*Gnaz*	464.46	150.21	433.1	505.83	467.72	3.11	guanine nucleotide binding protein, alpha z subunit
137133_s_at	*D930026N18Rik*	763.79	238.66	711	746.12	737.03	3.09	RIKEN cDNA D930026N18 gene
94802_at	*Mtss1l*	641.12	229.05	816.35	666.05	707.08	3.09	metastasis suppressor 1-like
166444_f_at	*Mfn2*	1214.13	369.49	1077.15	1042.7	1111.91	3.01	mitofusin 2

113708_at	*Endod1*	278.58	758.79	267.64	202.17	248.67	-3.05	endonuclease domain containing 1
95471_at	*Cdkn1c*	64.9	156.77	32.37	47.89	47.02	-3.33	cyclin-dependent kinase inhibitor 1C (P57)
113847_at	*Cdh4*	828.5	2713.76	605.02	969.66	801.73	-3.38	cadherin 4
93090_at	*Fgfr2*	516.25	1845.63	485.64	583.41	529.62	-3.48	fibroblast growth factor receptor 2
162941_at	*Usp25*	57.37	219.2	48.15	81.6	62.7	-3.50	ubiquitin specific peptidase 25
110399_at	*Srbd1*	420.22	1355.63	391.75	326.69	379	-3.58	S1 RNA binding domain 1
97834_g_at	*Pfkp*	162.48	758.12	255.54	198.28	210.96	-3.59	phosphofructokinase, platelet
108477_at	*Chsy1*	96.96	403.25	113.58	121.71	112.07	-3.60	chondroitin sulfate synthase 1
101502_at	*Tgif1*	89.8	286.93	72.95	66.87	76.13	-3.77	TGFB-induced factor homeobox 1
109421_at	*Spag5*	490.33	1355.78	233.78	353.74	359.75	-3.77	sperm associated antigen 5
107519_at	*Dock10*	191.39	597.61	157.87	127.83	157.23	-3.80	dedicator of cytokinesis 10
97168_at	*P2ry1*	206.57	1170.78	281.14	431.48	306.2	-3.82	purinergic receptor P2Y, G-protein coupled 1
111542_at	*Vps8*	765.79	2906.17	541.39	865.67	725.63	-4.01	vacuolar protein sorting 8 homolog (S. cerevisiae)
108806_r_at	*Arl4a*	34.64	141.92	36.78	33.77	35.04	-4.05	ADP-ribosylation factor-like 4A
113680_at	*Secisbp2l*	380.41	1525.68	361.05	371.09	371.55	-4.11	SECIS binding protein 2-like
111137_at	*Abcc4*	163.75	838.26	284.94	150.4	199.41	-4.20	ATP-binding cassette, sub-family C (CFTR/MRP), member 4
102726_at	*Tac1*	65.46	257.63	64.52	49	59.48	-4.33	tachykinin 1
99535_at	*Ccrn4l*	431.96	2964.63	1010.97	526.87	656.14	-4.52	CCR4 carbon catabolite repression 4-like (S. cerevisiae)
93316_at	*Osbpl1a*	235.23	957.5	271.17	128.17	211.37	-4.53	oxysterol binding protein-like 1A
161436_s_at	*Adarb1*	257.37	1100.41	216.38	225.41	232.5	-4.73	adenosine deaminase, RNA-specific, B1
104018_at	*Lct*	196.98	1923.57	435.02	569.28	400.9	-4.80	lactase
97504_at	*Ccnd2*	147.27	852.57	74.73	310.44	176.36	-4.83	cyclin D2
110457_at	*Hlf*	109.82	379.38	42.31	75.08	75.81	-5.00	hepatic leukemia factor
95608_at	*Ctsb*	30.82	199.43	65.76	21.34	39.46	-5.05	cathepsin B

135177_at	*Il13ra1*	147.59	883	92.87	259.84	166.84	-5.29	interleukin 13 receptor, alpha 1
163980_at	*Zfp185*	67.67	669.66	178.68	134.17	126.67	-5.29	zinc finger protein 185
95052_at	*Fam132a*	210.45	984.65	249.71	101.56	185.86	-5.30	family with sequence similarity 132, member A
100084_at	*Ezr*	273.67	1299.81	171.19	320.37	243.86	-5.33	ezrin
104412_at	*Gnai1*	154.93	1080.19	346.37	106.89	202.54	-5.33	guanine nucleotide binding protein (G protein), alpha inhibiting 1
116858_at	*Gnptab*	241.73	1234.19	229.08	221.88	231.4	-5.33	N-acetylglucosamine-1-phosphate transferase, alpha and beta subunits
92848_at	*Oat*	295.75	1753.26	608.13	79.17	327.59	-5.35	ornithine aminotransferase
167274_f_at	*Hspbp1*	22.31	603.62	185.25	93.07	106.83	-5.65	HSPA (heat shock 70kDa) binding protein, cytoplasmic cochaperone 1
103736_at	*Sash1*	84.34	566.6	152.55	58.51	99.67	-5.68	SAM and SH3 domain containing 1
94941_at	*Eif2ak4*	105.71	634.47	138.49	89.05	110.21	-5.76	eukaryotic translation initiation factor 2 alpha kinase 4
94633_at	*Gcg*	254.57	8993.26	2543.42	1871.81	1553.35	-5.79	glucagon
167130_r_at	*1810011O10Rik*	141.64	721.38	114.84	100.58	120.33	-5.99	RIKEN cDNA 1810011O10 gene
135069_at	*Bach2*	20.56	216.53	14.96	76.47	35.91	-6.03	BTB and CNC homology 2
102373_at	*Enpep*	90.34	757.04	178.1	101.71	123.65	-6.12	glutamyl aminopeptidase
117246_at	*Rpp25*	87.71	346.53	47.77	37.13	55.83	-6.21	ribonuclease P 25 subunit (human)
117310_at	*Sox11*	163.7	1180.66	206.66	201.34	188.51	-6.26	SRY-box containing gene 11
162969_at	*Edil3*	39.42	1365.87	404.84	178.09	207.34	-6.59	EGF-like repeats and discoidin I-like domains 3
92539_at	*S100a10*	592.13	4357.06	996.41	388.12	661.4	-6.59	S100 calcium binding protein A10 (calpactin)
98508_s_at	*Ppap2a*	141.11	1835.04	434.26	239.89	271.51	-6.76	phosphatidic acid phosphatase type 2A
166655_at	*Pcdh7*	3.5	357.12	110.54	51.6	51.96	-6.87	Protocadherin 7
107420_at	*Basp1*	9.67	153.9	17.09	39.24	21.52	-7.15	brain abundant, membrane attached signal protein 1
108749_at	*Tmcc3*	117.93	879.55	133.61	100.29	117.54	-7.48	transmembrane and coiled coil domains 3
113034_at	*Glyat*	23.5	204.47	41.39	15.37	26.91	-7.60	glycine-N-acyltransferase
115206_at	*Tbcel*	22.34	397.86	67.66	61.91	50.67	-7.85	tubulin folding cofactor E-like

102957_at	*Lcp2*	83.68	710.47	69.05	106.25	86.19	-8.24	lymphocyte cytosolic protein 2
163632_at	*Rbfox1*	59.33	491.02	62.72	43.23	54.53	-9.00	RNA binding protein, fox-1 homolog (C. elegans) 1
160083_at	*Lpl*	186.08	1474.6	160.85	136.78	160.58	-9.18	lipoprotein lipase
161132_at	*Scel*	11.32	316.25	68.01	17.64	30.47	-10.38	sciellin
104647_at	*Ptgs2*	15.32	195.77	26.11	11.41	18.23	-10.74	prostaglandin-endoperoxide synthase 2
92283_s_at	*Il4*	23.72	217.37	7.72	30.24	19.86	-10.94	interleukin 4
165640_at	*Prr5l*	90.54	969.2	66.24	82.09	81.07	-11.96	proline rich 5 like
99366_at	*Pqlc3*	164.07	2170.11	146.73	201.71	171.07	-12.69	PQ loop repeat containing
162171_f_at	*Fgfr2*	70.21	1035.33	34.31	92.4	66.58	-15.55	fibroblast growth factor receptor 2
103744_at	*Sh3bgrl2*	45.67	512.87	14.28	27.01	30.57	-16.78	SH3 domain binding glutamic acid-rich protein like 2
97825_at	*Perp*	96.03	997.97	37.53	20.48	52.66	-18.95	PERP, TP53 apoptosis effector
103723_at	*Il13ra1*	18.88	237.94	-54.36	60.93	11.1	-21.43	interleukin 13 receptor, alpha 1
115390_at	*Maf*	26.69	536.5	14.58	27.79	22.03	-24.36	avian musculoaponeurotic fibrosarcoma (v-maf) AS42 oncogene homolog
95670_at	*Stmn2*	45.02	1994.6	86.11	65.55	65.5	-30.45	stathmin-like 2
92642_at	*Car2*	84.97	2841.19	89.26	103.48	93	-30.55	carbonic anhydrase 2
103016_s_at	*Cd68*	51.68	2507.11	96.66	83.97	79.84	-31.40	CD68 antigen

*Probe names used in the murine genome U74 version 2 GeneChip array (Affymetrix).
**Raw values of expression intensities measured by Affymetrix arrays.
***Mean values of C4-LP, C4-HP, and Pr-LP.
****Ratio of responder mean to Pr-HP.
