# Supplementary material for: Microarray Analysis of Novel Candidate Genes Responsible for Glucose-Stimulated Insulin Secretion in Mouse Pancreatic β Cell Line MIN6
Source: PLoS One. 2013 Apr 3;8(4):e61211. doi: 10.1371/journal.pone.0061211 (PMC3616144; doi:10.1371/journal.pone.0061211)
Supplement: Table S3 — Genes differentially expressed between Pr-LP and clone 4 MIN6 cells. (PDF) [file pone.0061211.s004.pdf]

Table S3. Genes differentially expressed between Pr-LP and clone 4 MIN6 cells.

Probe*	Gene symbol	Pr-LP**	C4-LP**	C4-HP**	C4 mean***	Fold change****	Gene title
94545_at	Rtn1	72.63	948.92	490.22	719.57	9.91	reticulon 1
100050_at	Id1	121.13	925.61	1200.93	1063.27	8.78	inhibitor of DNA binding 1
166173_f_at	Lefty1	33.50	256.04	202.27	229.16	6.84	left right determination factor 1
104932_at	Slc2a13	39.01	329.76	176.51	253.13	6.49	solute carrier family 2 (facilitated glucose transporter), member 13
94633_at	Gcg	50.58	345.58	274.70	310.14	6.13	glucagon
111578_at	Tmem47	30.63	132.85	165.86	149.36	4.88	transmembrane protein 47
107884_at	Ttyh1	196.59	1277.38	638.86	958.12	4.87	tweety homolog 1 (Drosophila)
163581_at	Dclk1	25.95	154.78	95.04	124.91	4.81	doublecortin-like kinase 1
112322_at	AI662270	151.39	608.49	830.30	719.40	4.75	expressed sequence AI662270
164118_at	Foxp2	20.84	93.55	104.47	99.01	4.75	forkhead box P2
113970_at	Emilin1	203.05	1197.59	572.08	884.84	4.36	elastin microfibril interfacer 1
162969_at	Edil3	67.97	370.67	193.81	282.24	4.15	EGF-like repeats and discoidin I-like domains 3
101059_at	Ndn	171.00	734.05	585.14	659.59	3.86	necdin
135813_at	Tram1l1	41.51	192.83	126.83	159.83	3.85	translocation associated membrane protein 1-like 1
168147_s_at	Dclk1	31.47	122.09	104.12	113.10	3.59	doublecortin-like kinase 1
160190_at	Syt4	56.87	242.70	153.53	198.11	3.48	synaptotagmin IV
162573_at	Tmem59l	576.19	2323.68	1648.84	1986.26	3.45	transmembrane protein 59-like
165975_at	Stox2	38.43	155.60	105.58	130.59	3.40	storkhead box 2
97442_at	Slc39a8	119.67	357.35	455.49	406.42	3.40	solute carrier family 39 (metal ion transporter), member 8
134688_at	Foxp2	26.00	71.47	100.14	85.80	3.30	forkhead box P2
102414_i_at	Dnajc3	192.98	772.21	490.03	631.12	3.27	DnaJ (Hsp40) homolog, subfamily C, member 3
110314_at	Sdf2l1	617.56	2494.89	1482.99	1988.94	3.22	stromal cell-derived factor 2-like 1
107774_at	Tmem47	34.68	101.24	121.58	111.41	3.21	transmembrane protein 47
116425_at	Ntrk2	468.88	1748.59	1234.76	1491.67	3.18	neurotrophic tyrosine kinase, receptor, type 2
167783_f_at	Ubr4	692.71	2893.62	1459.36	2176.49	3.14	ubiquitin protein ligase E3 component n-recognin 4
92293_at	Nrcam	104.88	333.37	319.92	326.64	3.11	neuron-glia-CAM-related cell adhesion molecule
92248_at	Nr4a2	81.97	273.10	232.84	252.97	3.09	nuclear receptor subfamily 4, group A, member 2
94811_s_at	Ndn	320.95	1101.08	869.71	985.39	3.07	necdin
131107_at	Epb4.1l5	189.28	53.59	71.03	62.31	-3.04	erythrocyte protein band 4.1-like 5
139223_at	Rab37	376.97	127.11	120.80	123.96	-3.04	RAB37, member of RAS oncogene family
94350_f_at	Nqo1	135.62	42.22	44.99	43.60	-3.11	NAD(P)H dehydrogenase, quinone 1

139035_at	Tfrc	568.88	205.67	158.10	181.88	-3.13	transferrin receptor
113020_at	Atrnl1	200.03	67.72	58.17	62.94	-3.18	attractin like 1
94354_at	Abca1	495.44	163.71	139.02	151.37	-3.27	ATP-binding cassette, sub-family A (ABC1), member 1
109006_at	Epb4.1l5	375.13	92.22	134.32	113.27	-3.31	erythrocyte protein band 4.1-like 5
163127_at	Fam126a	263.21	80.40	77.55	78.97	-3.33	family with sequence similarity 126, member A
99972_at	Tph1	184.80	54.99	49.38	52.18	-3.54	tryptophan hydroxylase 1
96047_at	Rbp4	246.98	77.37	61.60	69.48	-3.55	retinol binding protein 4, plasma
167776_i_at	Syde2	253.76	64.68	77.50	71.09	-3.57	synapse defective 1, Rho GTPase, homolog 2 (C. elegans)
101441_i_at	Itpr2	175.48	55.74	39.85	47.79	-3.67	inositol 1,4,5-triphosphate receptor 2
92550_at	Krt19	223.38	57.58	56.42	57.00	-3.92	keratin 19
107952_i_at	Mreg	547.88	135.06	140.26	137.66	-3.98	melanoregulin
115070_at	Aqp4	281.71	78.77	48.01	63.39	-4.44	aquaporin 4
94351_r_at	Nqo1	301.93	50.05	71.81	60.93	-4.96	NAD(P)H dehydrogenase, quinone 1
103061_at	Gad1	231.41	46.07	45.56	45.82	-5.05	glutamate decarboxylase 1
169259_f_at	Six1	268.98	43.94	53.30	48.62	-5.53	sine oculis-related homeobox 1 homolog (Drosophila)
160702_at	Esrp1	251.49	43.15	45.42	44.28	-5.68	epithelial splicing regulatory protein 1
95661_at	Cd9	465.14	68.08	72.39	70.23	-6.62	CD9 antigen
100913_at	Fam151a	1224.59	149.12	150.60	149.86	-8.17	family with sequence simliarity 151, member A
165471_f_at	Arhgap36	122.75	15.67	11.94	13.80	-8.89	Rho GTPase activating protein 36

*Probe names used in the murine genome U74 version 2 GeneChip array (Affymetrix).

**Raw values of expression intensities measured by Affymetrix arrays.

***Mean values of C4-LP and C4-HP.

****Ratio of C4 mean to Pr-LP.
