# Supplementary material for: Microarray Analysis of Novel Candidate Genes Responsible for Glucose-Stimulated Insulin Secretion in Mouse Pancreatic β Cell Line MIN6
Source: PLoS One. 2013 Apr 3;8(4):e61211. doi: 10.1371/journal.pone.0061211 (PMC3616144; doi:10.1371/journal.pone.0061211)
Supplement: Table S4 — Genes differentially expressed between Pr-LP and C4-LP MIN6 cells. (PDF) [file pone.0061211.s005.pdf]

Table S4. Genes differentially expressed between Pr-LP and C4-LP MIN6 cells.

Probe*	Gene symbol	Pr-LP**	C4-LP**	Fold change***	Gene title
163627_f_at	Cthrc1	12.91	411.65	31.88	collagen triple helix repeat containing 1
166127_f_at	Gabrg2	8.06	217.88	27.04	gamma-aminobutyric acid (GABA) A receptor, subunit gamma 2
109769_f_at	Ttyh1	135.15	2120.26	15.69	tweety homolog 1 (Drosophila)
100477_at	Tmem45a	53.64	718.30	13.39	transmembrane protein 45a
94545_at	Rtn1	72.63	948.92	13.07	reticulon 1
101676_at	Gpx3	376.05	3890.37	10.35	glutathione peroxidase 3
104932_at	Slc2a13	39.01	329.76	8.45	solute carrier family 2 (facilitated glucose transporter), member 13
104464_s_at	Kdelr3	171.30	1409.57	8.23	KDEL (Lys-Asp-Glu-Leu) endoplasmic reticulum protein retention receptor 3
130122_f_at	Irx1	17.91	142.46	7.95	Iroquois related homeobox 1 (Drosophila)
166173_f_at	Lefty1	33.50	256.04	7.64	left right determination factor 1
100050_at	Id1	121.13	925.61	7.64	inhibitor of DNA binding 1
95350_at	Ttr	435.36	3134.52	7.20	transthyretin
107354_at	Slitrk5	226.72	1610.80	7.10	SLIT and NTRK-like family, member 5
94633_at	Gcg	50.58	345.58	6.83	glucagon
93285_at	Dusp6	182.29	1113.37	6.11	dual specificity phosphatase 6
109336_at	C1qtnf1	116.76	701.27	6.01	C1q and tumor necrosis factor related protein 1
163581_at	Dclk1	25.95	154.78	5.96	doublecortin-like kinase 1
102197_at	Nucb2	113.91	673.62	5.91	nucleobindin 2
113970_at	Emilin1	203.05	1197.59	5.90	elastin microfibril interfacer 1
101831_at	Shh	142.73	841.60	5.90	sonic hedgehog
99840_at	Pdyn	735.19	4296.67	5.84	prodynorphin
163173_at	Etv5	100.58	581.14	5.78	ets variant gene 5
99444_at	Ramp2	238.19	1364.64	5.73	receptor (calcitonin) activity modifying protein 2
112493_at	Ildr2	101.84	579.58	5.69	immunoglobulin-like domain containing receptor 2
112876_at	Diras2	103.96	575.88	5.54	DIRAS family, GTP-binding RAS-like 2
162969_at	Edil3	67.97	370.67	5.45	EGF-like repeats and discoidin I-like domains 3
98133_at	Calb1	44.05	225.63	5.12	calbindin 1
107622_at	Cdh13	99.99	508.70	5.09	cadherin 13
167769_f_at	Inpp5f	55.77	274.19	4.92	inositol polyphosphate-5-phosphatase F
161980_f_at	Bag3	83.89	394.03	4.70	BCL2-associated athanogene 3
135813_at	Tram1l1	41.51	192.83	4.65	translocation associated membrane protein 1-like 1
105224_at	Wipi1	424.09	1911.93	4.51	WD repeat domain, phosphoinositide interacting 1
97964_at	Fkbp11	397.73	1792.12	4.51	FK506 binding protein 11
168176_at	Slc17a9	158.83	696.89	4.39	solute carrier family 17, member 9
92378_at	Ptprz1	177.19	772.06	4.36	protein tyrosine phosphatase, receptor type Z, polypeptide 1
111578_at	Tmem47	30.63	132.85	4.34	transmembrane protein 47
101059_at	Ndn	171.00	734.05	4.29	necdin

160190_at	Syt4	56.87	242.70	4.27	synaptotagmin IV
128577_s_at	Cbln2	88.38	369.77	4.18	cerebellin 2 precursor protein
167783_f_at	Ubr4	692.71	2893.62	4.18	ubiquitin protein ligase E3 component n-recognin 4
102415_r_at	Dnajc3	53.69	220.55	4.11	DnaJ (Hsp40) homolog, subfamily C, member 3
160901_at	Fos	85.16	348.70	4.09	FBJ osteosarcoma oncogene
135902_at	Zcchc12	82.55	335.39	4.06	zinc finger, CCHC domain containing 12
116106_at	Btbd11	324.60	1316.07	4.05	BTB (POZ) domain containing 11
165975_at	Stox2	38.43	155.60	4.05	storkhead box 2
110314_at	Sdf2l1	617.56	2494.89	4.04	stromal cell-derived factor 2-like 1
162573_at	Tmem59l	576.19	2323.68	4.03	transmembrane protein 59-like
112322_at	AI662270	151.39	608.49	4.02	expressed sequence AI662270
102414_i_at	Dnajc3	192.98	772.21	4.00	DnaJ (Hsp40) homolog, subfamily C, member 3
165782_at	Ptprz1	68.93	275.35	3.99	protein tyrosine phosphatase, receptor type Z, polypeptide 1
116952_at	Pak3	52.93	209.31	3.95	p21 protein (Cdc42/Rac)-activated kinase 3
163396_at	Gria3	79.12	309.75	3.92	glutamate receptor, ionotropic, AMPA3 (alpha 3)
96072_at	Ldha	358.30	1396.83	3.90	lactate dehydrogenase A
164295_i_at	Nceh1	137.01	531.72	3.88	arylacetamide deacetylase-like 1
168147_s_at	Dclk1	31.47	122.09	3.88	doublecortin-like kinase 1
103891_i_at	Ell2	496.18	1887.39	3.80	elongation factor RNA polymerase II 2
113335_at	Fam46a	628.34	2385.95	3.80	family with sequence similarity 46, member A
98983_at	P4ha2	366.06	1382.41	3.78	procollagen-proline, 2-oxoglutarate 4-dioxygenase, alpha II polypeptide
104139_at	P4ha1	131.66	495.04	3.76	procollagen-proline, 2-oxoglutarate 4-dioxygenase, alpha 1 polypeptide
104041_at	Ubr4	2997.19	11240.13	3.75	ubiquitin protein ligase E3 component n-recognin 4
116425_at	Ntrk2	468.88	1748.59	3.73	neurotrophic tyrosine kinase, receptor, type 2
96679_at	Dnajb9	431.85	1609.17	3.73	DnaJ (Hsp40) homolog, subfamily B, member 9
112922_i_at	Kdelr3	99.31	366.98	3.70	KDEL (Lys-Asp-Glu-Leu) endoplasmic reticulum protein retention receptor 3
101571_g_at	Igfbp4	196.94	715.41	3.63	insulin-like growth factor binding protein 4
135776_at	Pcdh11x	42.73	151.80	3.55	protocadherin 11 X-linked
116877_at	Vgf	2574.72	9144.68	3.55	VGF nerve growth factor inducible
116405_at	Tmem179	470.33	1659.48	3.53	transmembrane protein 179
99623_s_at	Olfm1	112.34	389.96	3.47	olfactomedin 1
163116_at	Vav3	120.48	416.26	3.46	vav 3 oncogene
104422_at	Ptprn	3637.53	12536.05	3.45	protein tyrosine phosphatase, receptor type, N
166303_i_at	Wnt5a	41.07	140.98	3.43	wingless-related MMTV integration site 5A
94811_s_at	Ndn	320.95	1101.08	3.43	necdin
93214_at	Camk2d	36.24	124.03	3.42	calcium/calmodulin-dependent protein kinase II, delta
104882_at	Prlr	52.07	177.57	3.41	prolactin receptor
95620_at	Dhrs7	266.20	906.74	3.41	dehydrogenase/reductase (SDR family) member 7
103394_at	Fxyd5	145.77	494.79	3.39	FXYD domain-containing ion transport regulator 5
101963_at	Ctsl	1050.80	3560.96	3.39	cathepsin L
106198_at	Vash2	96.53	326.52	3.38	vasohibin 2

92210_at	Angpt2	58.71	197.37	3.36	angiopoietin 2
92248_at	Nr4a2	81.97	273.10	3.33	nuclear receptor subfamily 4, group A, member 2
98572_at	Dnajb11	171.95	570.89	3.32	DnaJ (Hsp40) homolog, subfamily B, member 11
160386_at	Clptm1l	721.97	2386.67	3.31	CLPTM1-like
134046_at	Wnt5a	61.13	202.04	3.30	wingless-related MMTV integration site 5A
93155_at	Frmd5	94.92	312.93	3.30	FERM domain containing 5
102389_s_at	Gap43	118.55	388.80	3.28	growth associated protein 43
96167_at	Bag3	268.14	874.24	3.26	BCL2-associated athanogene 3
104143_at	Copz2	414.96	1346.58	3.25	coatomer protein complex, subunit zeta 2
162927_at	Dner	485.00	1571.15	3.24	delta/notch-like EGF-related receptor
162739_at	Inpp5f	31.93	101.91	3.19	inositol polyphosphate-5-phosphatase F
105240_at	Pde1c	94.79	302.29	3.19	phosphodiesterase 1C
92293_at	Nrcam	104.88	333.37	3.18	neuron-glia-CAM-related cell adhesion molecule
160715_at	Vopp1	73.51	233.39	3.17	vesicular, overexpressed in cancer, prosurvival protein 1
164417_f_at	Fkbp11	212.94	672.47	3.16	FK506 binding protein 11
94207_at	Pdia6	317.56	1002.36	3.16	protein disulfide isomerase associated 6
164014_at	Fam114a1	101.61	318.38	3.13	family with sequence similarity 114, member A1
92540_f_at	Srm	698.81	2185.34	3.13	spermidine synthase
164060_at	Arf4	41.81	130.62	3.12	ADP-ribosylation factor 4
163745_at	Yipf6	417.92	1298.49	3.11	Yip1 domain family, member 6
96122_at	Cmbl	286.27	887.00	3.10	carboxymethylenebutenolidase-like (Pseudomonas)
168114_i_at	Syt4	1608.55	4983.37	3.10	synaptotagmin IV
103892_r_at	Ell2	101.00	311.51	3.08	elongation factor RNA polymerase II 2
99144_s_at	Tgoln1 /// Tgoln2	346.42	1064.85	3.07	trans-golgi network protein /// trans-golgi network protein 2
93497_at	C3 /// LOC10004875	112.66	344.62	3.06	complement component 3 /// complement C3-like
165691_at	Pycr1	667.20	2033.49	3.05	pyrroline-5-carboxylate reductase 1
140885_at	Ntrk2	134.23	406.95	3.03	neurotrophic tyrosine kinase, receptor, type 2
168489_r_at	Cacng2	50.85	154.00	3.03	calcium channel, voltage-dependent, gamma subunit 2
104065_at	Edem1	79.92	240.51	3.01	ER degradation enhancer, mannosidase alpha-like 1
100771_at	Blnk	1531.02	4592.18	3.00	B cell linker
99032_at	Rasd1	349.01	115.43	-3.02	RAS, dexamethasone-induced 1
94354_at	Abca1	495.44	163.71	-3.03	ATP-binding cassette, sub-family A (ABC1), member 1
136586_at	Arpc1b /// Gm5637	221.83	73.21	-3.03	actin related protein 2/3 complex, subunit 1B /// predicted pseudogene 5637
94148_at	Ppy	360.60	118.19	-3.05	pancreatic polypeptide
169294_s_at	Myt1	347.39	113.31	-3.07	myelin transcription factor 1
103888_at	Rbpms	914.88	296.52	-3.09	RNA binding protein gene with multiple splicing
92500_at	Odz3	281.77	90.65	-3.11	odd Oz/ten-m homolog 3 (Drosophila)
164137_at	Cdhr1	1903.35	611.15	-3.11	cadherin-related family member 1
101441_i_at	Itpr2	175.48	55.74	-3.15	inositol 1,4,5-triphosphate receptor 2
101828_at	Ret	283.79	90.05	-3.15	ret proto-oncogene
96047_at	Rbp4	246.98	77.37	-3.19	retinol binding protein 4, plasma

94350_f_at	Nqo1	135.62	42.22	-3.21	NAD(P)H dehydrogenase, quinone 1
114696_at	Klf11	513.15	157.36	-3.26	Kruppel-like factor 11
163127_at	Fam126a	263.21	80.40	-3.27	family with sequence similarity 126, member A
112386_at	Cald1	117.43	35.74	-3.29	caldesmon 1
95485_at	Hadh	2320.35	703.94	-3.30	hydroxyacyl-Coenzyme A dehydrogenase
99972_at	Tph1	184.80	54.99	-3.36	tryptophan hydroxylase 1
116165_at	Fam210b	1044.08	306.65	-3.40	family with sequence similarity 210, member B
94057_g_at	Scd1	1328.71	385.30	-3.45	stearoyl-Coenzyme A desaturase 1
131107_at	Epb4.1l5	189.28	53.59	-3.53	erythrocyte protein band 4.1-like 5
97393_at	Vrk1	507.60	143.49	-3.54	vaccinia related kinase 1
166432_f_at	H2-Q2	1435.93	402.92	-3.56	histocompatibility 2, Q region locus 2
115070_at	Aqp4	281.71	78.77	-3.58	aquaporin 4
161026_s_at	Sytl4	1538.71	426.81	-3.61	synaptotagmin-like 4
171190_f_at	Odz3	667.50	181.90	-3.67	odd Oz/ten-m homolog 3 (Drosophila)
110056_at	Acvr1c	132.68	35.75	-3.71	activin A receptor, type IC
103222_at	Eps8	170.81	45.72	-3.74	epidermal growth factor receptor pathway substrate 8
92550_at	Krt19	223.38	57.58	-3.88	keratin 19
167776_i_at	Syde2	253.76	64.68	-3.92	synapse defective 1, Rho GTPase, homolog 2 (C. elegans)
97206_at	Spint1	400.75	102.07	-3.93	serine protease inhibitor, Kunitz type 1
136724_at	Scgn	4282.53	1081.71	-3.96	secretagogin, EF-hand calcium binding protein
165621_at	1700086L19Rik	176.35	43.70	-4.04	RIKEN cDNA 1700086L19 gene
107952_i_at	Mreg	547.88	135.06	-4.06	melanoregulin
109006_at	Epb4.1l5	375.13	92.22	-4.07	erythrocyte protein band 4.1-like 5
133951_at	Ky	525.58	120.95	-4.35	kyphoscoliosis peptidase
114557_at	Phf16	336.64	69.88	-4.82	PHD finger protein 16
103061_at	Gad1	231.41	46.07	-5.02	glutamate decarboxylase 1
160702_at	Esrp1	251.49	43.15	-5.83	epithelial splicing regulatory protein 1
94351_r_at	Nqo1	301.93	50.05	-6.03	NAD(P)H dehydrogenase, quinone 1
169259_f_at	Six1	268.98	43.94	-6.12	sine oculis-related homeobox 1 homolog (Drosophila)
95661_at	Cd9	465.14	68.08	-6.83	CD9 antigen
113219_at	Cpm	137.33	18.65	-7.36	carboxypeptidase M
109069_at	Slc40a1	1823.67	244.81	-7.45	solute carrier family 40 (iron-regulated transporter), member 1
165471_f_at	Arhgap36	122.75	15.67	-7.84	Rho GTPase activating protein 36
100913_at	Fam151a	1224.59	149.12	-8.21	family with sequence simliarity 151, member A
160074_at	Ddc	4386.79	150.45	-29.16	dopa decarboxylase

*Probe names used in the murine genome U74 version 2 GeneChip array (Affymetrix).

**Raw values of expression intensities measured by Affymetrix arrays.

***Ratio of C4-LP to Pr-LP.
