# Supplementary material for: Microarray Analysis of Novel Candidate Genes Responsible for Glucose-Stimulated Insulin Secretion in Mouse Pancreatic β Cell Line MIN6
Source: PLoS One. 2013 Apr 3;8(4):e61211. doi: 10.1371/journal.pone.0061211 (PMC3616144; doi:10.1371/journal.pone.0061211)
Supplement: Table S5 — Genes differentially expressed between C4-LP and C4-HP MIN6 cells. (PDF) [file pone.0061211.s006.pdf]

Table S5. Genes differentially expressed between C4-LP and C4-HP MIN6 cells.

Probe*	Gene symbol	C4-LP**	C4-HP**	Fold change***	Gene title
163627_f_at	Cthrc1	411.65	12.65	32.54	collagen triple helix repeat containing 1
166127_f_at	Gabrg2	217.88	11.74	18.55	gamma-aminobutyric acid (GABA) A receptor, subunit gamma 2
101676_at	Gpx3	3890.37	276.35	14.08	glutathione peroxidase 3
109336_at	C1qtnf1	701.27	85.61	8.19	C1q and tumor necrosis factor related protein 1
97519_at	Spp1	798.10	109.59	7.28	secreted phosphoprotein 1
130122_f_at	Irx1	142.46	22.82	6.24	Iroquois related homeobox 1 (Drosophila)
166874_r_at	Ildr2	776.00	129.91	5.97	immunoglobulin-like domain containing receptor 2
96605_at	Tmem176a	1254.20	251.36	4.99	transmembrane protein 176A
107622_at	Cdh13	508.70	103.69	4.91	cadherin 13
99065_at	Csn3	847.02	175.98	4.81	casein kappa
165782_at	Ptprz1	275.35	57.91	4.75	protein tyrosine phosphatase, receptor type Z, polypeptide 1
112876_at	Diras2	575.88	122.20	4.71	DIRAS family, GTP-binding RAS-like 2
163646_at	Jam2	140.39	30.22	4.65	junction adhesion molecule 2
136702_at	C030034E14Rik	1789.56	386.66	4.63	RIKEN cDNA C030034E14 gene
93285_at	Dusp6	1113.37	246.64	4.51	dual specificity phosphatase 6
109403_at	Cd44	420.53	95.39	4.41	CD44 antigen
105224_at	Wipi1	1911.93	448.47	4.26	WD repeat domain, phosphoinositide interacting 1
116952_at	Pak3	209.31	49.96	4.19	p21 protein (Cdc42/Rac)-activated kinase 3
128577_s_at	Cbln2	369.77	89.07	4.15	cerebellin 2 precursor protein
135902_at	Zcchc12	335.39	81.06	4.14	zinc finger, CCHC domain containing 12
98133_at	Calb1	225.63	55.47	4.07	calbindin 1
92378_at	Ptprz1	772.06	194.89	3.96	protein tyrosine phosphatase, receptor type Z, polypeptide 1
114697_at	Cd44	350.78	88.62	3.96	CD44 antigen
106061_at	Mmd2	1071.56	271.30	3.95	monocyte to macrophage differentiation-associated 2
161980_f_at	Bag3	394.03	100.69	3.91	BCL2-associated athanogene 3
115859_at	Ldlrad3	484.89	124.96	3.88	low density lipoprotein receptor class A domain containing 3
102815_at	Anxa11 /// Gm2260 /// Gm2274	398.20	102.68	3.88	annexin A11 /// predicted gene 2260 /// predicted gene 2274
103394_at	Fxyd5	494.79	130.10	3.80	FXYD domain-containing ion transport regulator 5
113651_at	Pir	467.14	124.28	3.76	pirin
105871_at	Dusp4	971.14	260.97	3.72	dual specificity phosphatase 4
100771_at	Blnk	4592.18	1259.58	3.65	B cell linker
106198_at	Vash2	326.52	91.45	3.57	vasohibin 2
163013_at	Shisa2	177.08	50.01	3.54	shisa homolog 2 (Xenopus laevis)
92210_at	Angpt2	197.37	56.06	3.52	angiopoietin 2
94432_at	St6gal1	301.63	86.41	3.49	beta galactoside alpha 2,6 sialyltransferase 1
113335_at	Fam46a	2385.95	695.04	3.43	family with sequence similarity 46, member A
99045_at	Eno2	999.41	296.00	3.38	enolase 2, gamma neuronal

101963_at	Ctsl	3560.96	1062.56	3.35	cathepsin L
92665_f_at	3830403N18Rik /// Xlr	315.13	96.23	3.27	RIKEN cDNA 3830403N18 gene /// X-linked lymphocyte-regulated complex
163574_at	Slc4a10	981.36	304.18	3.23	solute carrier family 4, sodium bicarbonate cotransporter-like, member 10
99504_at	St8sia3	514.73	160.48	3.21	ST8 alpha-N-acetyl-neuraminide alpha-2,8-sialyltransferase 3
92880_at	Mfge8	4438.05	1399.68	3.17	milk fat globule-EGF factor 8 protein
102805_at	Ceacam1	728.88	230.13	3.17	carcinoembryonic antigen-related cell adhesion molecule 1
103676_at	C1qtnf1	390.69	124.62	3.13	C1q and tumor necrosis factor related protein 1
96336_at	Gatm	1210.02	387.64	3.12	glycine amidinotransferase (L-arginine:glycine amidinotransferase)
100477_at	Tmem45a	718.30	230.26	3.12	transmembrane protein 45a
101571_g_at	Igfbp4	715.41	229.56	3.12	insulin-like growth factor binding protein 4
93497_at	C3 /// LOC100048759	344.62	110.72	3.11	complement component 3 /// complement C3-like
164014_at	Fam114a1	318.38	103.49	3.08	family with sequence similarity 114, member A1
116114_at	Wipi1	714.47	232.85	3.07	WD repeat domain, phosphoinositide interacting 1
160185_at	Tagln3	564.92	185.27	3.05	transgelin 3
100772_g_at	Blnk	1453.77	477.65	3.04	B cell linker
100690_at	Th	2180.97	719.15	3.03	tyrosine hydroxylase
161026_s_at	Sytl4	426.81	1315.31	-3.08	synaptotagmin-like 4
99833_at	Capn9	146.13	451.92	-3.09	calpain 9
167874_at	Pabpc1l	128.04	399.07	-3.12	poly(A) binding protein, cytoplasmic 1-like
114557_at	Phf16	69.88	218.57	-3.13	PHD finger protein 16
166517_f_at	Alcam	80.17	255.85	-3.19	activated leukocyte cell adhesion molecule
93403_at	Atp2a3	223.17	712.91	-3.19	ATPase, Ca++ transporting, ubiquitous
102926_at	Gfra3	372.20	1192.32	-3.20	glial cell line derived neurotrophic factor family receptor alpha 3
96203_at	Calml4	157.11	504.49	-3.21	calmodulin-like 4
103714_at	Iyd	73.89	243.59	-3.30	iodotyrosine deiodinase
110056_at	Acvr1c	35.75	120.58	-3.37	activin A receptor, type IC
160934_s_at	Sgip1	2093.62	7387.79	-3.53	SH3-domain GRB2-like (endophilin) interacting protein 1
165910_f_at	Upk3a	317.49	1129.35	-3.56	uroplakin 3A
103357_at	Slc2a2	463.81	1754.45	-3.78	solute carrier family 2 (facilitated glucose transporter), member 2
162973_at	Nefl	430.84	1637.48	-3.80	neurofilament, light polypeptide
168250_f_at	Gm5465	160.60	636.80	-3.97	predicted gene 5465
102348_at	Gm4354	99.96	469.96	-4.70	predicted gene 4354
167905_f_at	Flrt3	79.45	415.63	-5.23	fibronectin leucine rich transmembrane protein 3
160074_at	Ddc	150.45	988.24	-6.57	dopa decarboxylase
115520_at	Trim12c /// Trim5	52.66	584.21	-11.09	tripartite motif-containing 12C /// tripartite motif-containing 5

*Probe names used in the murine genome U74 version 2 GeneChip array (Affymetrix).
**Raw values of expression intensities measured by Affymetrix arrays.
***Ratio of C4-LP to C4-HP.
